# *Bacillus subtilis* and chitosan nanoparticles enhance potato virus Y (PVY) tolerance in tomato* (Solanum lycopersicum L.)* via modulation of antioxidants and secondary metabolites

**DOI:** 10.1186/s12870-025-07617-0

**Published:** 2025-11-20

**Authors:** Amira M. Ghanaim, Ghada A. Mahmoud, Heba I. Mohamed, Rania S. Hanafy, Laila M. Zaki, Mohamed Mahmoud, Asmaa M. Mogazy

**Affiliations:** 1 Department of Biological and Geological Sciences, Faculty of Education, Ain Shams University, Cairo, 11341 Egypt; 2 Department of Agricultural Biochemistry, Faculty of Agriculture, Ain Shams University, Hadayek Shobra, Cairo, 11241 Egypt

**Keywords:** Enzymatic and non-enzymatic antioxidant, Antiviral activity, Oxidative stress, Osmoprotectants, Secondary metabolites, Yield components

## Abstract

**Background:**

Plant viral infections threaten global food security and cause major crop losses. This study investigates the antiviral effects of biosynthesized Chitosan nanoparticles (ChNPs) with *Bacillus subtilis* 1211 EMCCN against potato virus Y (PVY) and their influence on tomato growth and yield.

**Results:**

Scanning electron microscopy showed clusters of crystals on ChNPs, while transmission electron microscopy revealed that the nanoparticles were spherical, ranging from 12 to 198 nm. Energy-dispersive X-ray spectroscopy confirmed the presence of carbon, oxygen, sodium, and phosphorus, and Fourier-transform infrared analysis identified typical chitosan functional groups, including hydroxyl, carbonyl, and amine. Tomato leaves were treated with ChNPs, *B. subtilis*, or both to evaluate their effectiveness against PVY. The treated plants exhibited a marked reduction in both disease severity and PVY concentration compared to the untreated controls. By 28 days post-inoculation, infectivity decreased to 46.7% in plants treated with Bacillus subtilis, 33.3% with ChNPs, and reached the lowest level of 20% in the combined treatment. The combined priming strategy significantly enhanced plant growth attributes, with increases in shoot length (95.0%), root length (47.0%), leaf area (668.1%), plant height (30.7%), shoot and root fresh weight (370.0% and 162.9%), and shoot and root dry weight (562.7% and 127.7%). Biochemical and physiological analyses revealed substantial increases in total pigments (268.4%), flavonoids (112.2%), phenols (59.4%), α-tocopherol (92.1%), ascorbic acid (30.1%), anthocyanins (71.3%), peroxidase (51.9%), catalase (39.6%), polyphenol oxidase (73.6%), amino acids (119.9%), proline (93.3%), soluble sugars (128.0%), and proteins (614.0%) in shoots. Yield components, including carotenoids and anthocyanins, were also significantly enhanced. Importantly, oxidative stress indicators were markedly reduced, with malondialdehyde and hydrogen peroxide levels decreasing by 76.8% and 72.6%, respectively, in treated plants compared to infected plants.

**Conclusion:**

These findings suggest that combination between ChNPs and *B. subtilis* offers an eco-friendly method to enhance tomato yield and effectively manage viral diseases by activating the plants' defense mechanisms.

## Introduction

The growing global population, along with urbanization and climate change, is driving an increasing demand for higher crop yields and better food quality. Various strategies are being implemented to accelerate and enhance agricultural production. However, plant diseases significantly contribute to crop losses and hinder progress in crop management [1]. Among these, viral diseases present major threats to global agriculture, affecting a wide range of economically important crops. These diseases can lead to severe yield losses, decreased crop quality, and higher production costs, ultimately impacting food security and the livelihoods of farmers [2]. The genus Potyvirus, part of the family Potyviridae, is one of the largest groups of plant RNA viruses and infects a diverse array of commercially important agricultural crops [3]. Potato virus Y (PVY) is a highly destructive plant pathogen from the genus Potyvirus. It ranks among the most economically significant viruses, leading to severe yield losses in important Solanaceae crops, such as potatoes, tomatoes, peppers, and tobacco [4, 5]. The virus spreads through infected planting material and aphid vectors in a non-persistent manner, complicating control measures [4]. Additionally, PVY can be transmitted mechanically or through infected plants, posing a continued challenge for tomato growers, especially in areas with mixed cropping systems or nearby infected potato and tobacco fields. As a result, PVY is considered the most harmful virus affecting tomato and other Solanaceous crop production areas [6].

The tomato (*Solanum lycopersicum*) is one of the most widely cultivated vegetable crops globally, valued for its nutritional benefits and economic importance [7]. However, tomato production faces significant challenges due to various plant pathogens, particularly viruses. These viruses are particularly harmful because they can spread rapidly and persist within agricultural systems [8]. Viral diseases in tomatoes can result in severe yield losses, reduced fruit quality, and increased production costs, posing a substantial threat to food security and the livelihoods of farmers worldwide [9]. For example, infection with Potato Virus Y (PVY) can lead to symptoms such as leaf mottling, mosaic patterns, leaf curling, deformation, stunting, and, in some cases, malformation or necrosis of the fruit [9].

Currently, most concern regarding tomato viral diseases in the Middle East focuses on *Tomato yellow leaf curl virus* (TYLCV) and *Tomato brown rugose fruit virus* (ToBRFV). However, *Potato virus Y* (PVY) also poses a significant, though often underrecognized, economic threat. TYLCV and ToBRFV have garnered major attention because early infections can lead to yield losses of 20–100% and 15–70%, respectively. Their rapid spread through whiteflies or mechanical contact has resulted in devastating regional outbreaks. In contrast, PVY infections in tomatoes can cause yield reductions ranging from 39 to 75%, with severe cases resulting in losses approaching 80%. These variations depend on factors such as strain virulence, cultivar susceptibility, and timing of infection [8, 10, 11]. Field surveys and experimental studies indicate that yield losses of 10–53% can occur even at moderate infection levels, leading to significant declines in both marketable fruit and seed quality [12–15]. Although PVY has traditionally been studied in potatoes, its increasing detection in tomato fields across Europe, North Africa, and the Middle East suggests it could substantially contribute to production instability, particularly where mixed infections with TYLCV or tobamoviruses are present. Given that PVY is efficiently transmitted by aphids in a non-persistent manner, its potential for rapid spread under high vector pressure underscores the importance of including it in regional tomato virus management programs [15, 16]. In Egypt several viral and viral-like agents were identified recently affecting tomato crops and lead to considerable yield losses and include *Tomato yellow leaf curl virus* (TYLCV), *Tomato ringspot virus* (ToRSV), *Cucumber mosaic virus* (CMV), *Tobacco mosaic virus* (TMV), *Tomato bushy stunt virus* (TBSV), *Tomato spotted wilt virus* (TSWV) and *Potato virus Y* (PVY) [8, 16].

Controlling the economic damage caused by viral pathogens is a critical issue in 21st-century agriculture. Many farmers around the world depend on chemical pesticides and fertilizers to achieve high agricultural yields [10]. However, these chemical antiviral compounds can be toxic to plants, animals, and humans. Classical antiviral agents, such as ribavirin (1-β-D-ribofuranosyl-1,2,4-triazole-3-carboxamide), thiouracil, and benzimidazole derivatives, can inhibit virus replication by interfering with nucleic acid synthesis. However, these compounds also disrupt normal plant metabolism. For instance, ribavirin, a purine analogue effective against the TMV and PVY in experimental settings, often leads to chlorosis, stunted growth, and necrotic lesions. These effects arise from its interference with RNA synthesis and plant enzyme systems [17, 18]. Similarly, thiouracil and related uracil analogues inhibit pyrimidine metabolism, resulting in leaf deformation, reduced chlorophyll content, and growth retardation when used at concentrations that are effective for viral suppression [19]. Additionally, developing virus-resistant plants through genetic transformation can be costly, time-consuming, and limited in its applications [20]. As a result, there is increasing interest in environmentally friendly methods for protecting plants from viruses. This includes the use of plant growth-promoting rhizobacteria (PGPR), such as *Bacillus subtilis*, as well as the exploration of nanoparticles as new antiviral agents and biological triggers [21, 22].

Plant growth-promoting *Bacillus subtilis* has a unique ability to replicate quickly, resist harsh environmental conditions, and provide broad biological control [10]. Additionally, the volatile compounds produced by *B. subtilis* play a vital role in supporting plant growth and activating plant defense mechanisms through a process known as induced systemic resistance (ISR) [1]. *Bacillus subtilis* can prime a host plant's immune system to better resist viral infections by inducing Systemic Acquired Resistance (SAR) and Induced Systemic Resistance (ISR). The bacteria achieve this by producing compounds like lipopeptides, which activate defense-related genes and signaling pathways involving plant hormones such as salicylic acid and jasmonic acid, preparing the plant to mount a faster and stronger defense against subsequent viral attacks [23]. Furthermore, it has been demonstrated that specific strains of *B. subtilis* create lipopeptides like surfactin, iturin, and fengycin that disrupt the viral infection process and prevent the spread of viruses within host tissues [24]. In addition to their antibacterial qualities, these metabolites also stimulate plant defense mechanisms, strengthening defenses against viruses such as PVY and other pathogens [25]. *B. subtilis* provides a complete solution for sustainable crop management under viral load since, in addition to its direct antiviral actions, it also grows roots and shoots, enhances stress tolerance, and improves nutrient uptake.

In recent years, nanotechnology has become a promising method for plant protection, particularly through the use of chitosan nanoparticles (ChNPs). These nanoparticles exhibit both antiviral activity and the ability to stimulate plant immune responses. Chitosan, a natural polysaccharide derived from chitin, is biodegradable, biocompatible, and has intrinsic antimicrobial properties [26]. CNPs enhance plant uptake, surface activity, and interactions with plant cells and pathogens due to their small size, increased surface area, and ability to traverse biological barriers like plant cell walls. This makes them an effective tool in agriculture for delivering agrochemicals, improving plant growth, and protecting against pests and diseases by acting as nanocarriers and inducing plant defense mechanisms [27]. Chitosan nanoparticles have shown significant antiviral effects by interfering with virus replication, movement, and the development of symptoms [28].

Research has shown that treating plants infected with virus with chitosan nanoparticles can significantly reduce viral load, inhibit systemic infection, and minimize the expression of disease symptoms [29]. This antiviral effect is partially due to the induction of systemic resistance in the host plant. Chitosan nanoparticles enhance the plant's innate immunity by strengthening the physical barriers of the cell walls and increasing the activity of enzymes involved in oxidative stress responses [30]. Their use not only suppresses viral multiplication but also improves the overall vigor of the plants and their tolerance to biotic stress. These numerous benefits make chitosan nanoparticles an environmentally friendly and effective alternative to conventional chemicals for managing viral diseases like PVY [31, 32]. As research continues, optimizing the formulations and delivery methods of these nanoparticles will be essential for maximizing their protective effects in crop management strategies.

The objective of this study is to evaluate the efficacy of *Bacillus subtilis* (Bs), chitosan nanoparticles (ChNPs), and their combined application as pre-infection treatments for the biological control of Potato virus Y (PVY) in tomato plants, with the aim of enhancing plant resistance, reducing viral incidence, and promoting overall plant health. The study also aims to measure virus concentration, assess disease severity, and examine various vegetative and yield attributes. Additionally, it will analyze photosynthetic pigments, osmolytes, secondary metabolites, and both enzymatic and non-enzymatic antioxidants, along with the nutritional content of the tomato fruits.

## Materials and methods

### Plant material

*S. lycopersicum* (strain 010) tomato seedlings were the experimental plants employed in this study. The Ministry of Agriculture Research Center in Giza, Egypt, provided the pure strain of seedlings. After preliminary studies, the tested strain exhibits a *susceptible response* to PVY, characterized by pronounced leaf mottling and yield suppression.

### Source of plant growth-promoting rhizobacteria

In this investigation, *B. subtilis* (1211 EMCCN) isolates were obtained from the Egyptian Microbial Culture Collection (EMCCN), which is located at Ain Shams University. The strain was kept in nutrient-agar growth media under carefully monitored circumstances.

### Sample collection and virus detection

A naturally occurring tomato that exhibited symptoms similar to a virus was collected from the Qalubia Governorate in Egypt. It was thought that PVY was responsible for these symptoms. Forty tomato plant samples exhibiting symptoms were collected, along with ten leaf samples that showed no symptoms. Stunted growth, leaf distortion, mosaic patterns, and mottling are typical symptoms. To maintain viral integrity throughout transportation to the lab, samples are gathered in sterile plastic bags and kept on ice. After that, leaves are processed within 24 to 48 h and kept at 4 °C to stop the viral RNA from degrading [33, 34].

### Isolation and Propagation of PVY

As an abrasive, 1–2 g of diseased leaf tissue are pulverized in phosphate buffer (0.01 M, pH 7.0) with 0.1% sodium sulfite and a tiny bit of carborundum (600 mesh) for mechanical sap inoculation. *Datura metel, Solanum lycopersicum*, and *Solanum tuberosum* are examples of healthy indicator plants whose leaves are gently rubbed with the resultant homogenate. These plants are known to show distinct symptoms when infected with PVY. Plants are kept in a greenhouse with a 16-h photoperiod and 22–25 °C. They are monitored every day for the onset of symptoms for two to three weeks [35]. The PVY inoculum was prepared from infected tomato leaves by homogenizing the tissue in a 1:10 (w/v) ratio with 0.01 M phosphate buffer (pH 7.2) that contained 0.1% sodium sulfite. The homogenate was then filtered and used immediately for mechanical inoculation. Before inoculation, each test plant was lightly dusted with carborundum (600 mesh). Approximately 1 mL of the viral inoculum was applied to each of two leaves per plant. The viral suspension had an optical density of 0.7 at 260 nm, which confirmed that there was an adequate viral concentration for consistent infection.

### Serological identification of PVY

The samples were analyzed using DAS-ELISA with PVY polyclonal antibody, as explained by Clark and Adams [33]. In this paper, anti-PVY antibody-coated microtiter plates are supplemented with leaf extracts. Following washing, substrate development (often p-nitrophenyl phosphate or TMB) is followed by the addition of enzyme-conjugated secondary antibodies. Samples with results over the threshold (negative controls + 3 × standard deviation) are regarded as positive for PVY. Absorbance is evaluated at 405 nm.

## Molecular identification

### Molecular Detection of PVY by RT-PCR

Total RNA was extracted from 100 mg of tomato leaf tissue using the Plant Total RNA Mini Kit (RBC Labs) according to the manufacturer’s protocol. The leaf tissue was ground in liquid nitrogen, mixed with RB buffer and β-mercaptoethanol, and clarified via filtration and ethanol precipitation. The RNA was then purified through spin-column washes and eluted in RNase-free water. RNA quality and concentration were assessed spectrophotometrically (A260/A280) and verified by 1% agarose gel electrophoresis. For PVY detection, a one-step RT-PCR was performed with the Verso 1-Step RT-PCR Kit (Thermo Scientific) using gene-specific primers designed by Shalaby et al. [36]. The thermal cycling profile included reverse transcription at 50 °C for 30 min, enzyme inactivation at 94 °C for 2 min, followed by 35 cycles of denaturation at 94 °C, annealing at 55 °C, and extension at 72 °C, each for 1 min, and a final extension at 72 °C for 5 min. The amplified products (5 µL) were analyzed by electrophoresis on a 1% agarose gel in TBE buffer, stained with ethidium bromide, and visualized under UV light using a Bio-Rad GelDoc XR system. A 100 bp DNA ladder (RBC) served as a size marker [37].

### Chitosan nanoparticles green synthesis

To remove contaminants and undesired components, the resistant tomato fruits cultivars to PVY (Strain 186) were cleaned three times with tap water and then once with distilled water. Fifty grams of plant material were then chopped into little pieces and forcefully mixed with five hundred milliliters of distilled water. To get a pure extract devoid of impurities, the extract was subsequently filtered through filter paper [38].

ChNPs were synthesized using the method described by Abdallah et al. [38]. In summary, a chitosan-TPP mixture was prepared by dissolving 0.8 g of sodium tripolyphosphate (TPP) from Aladdin Industrial Co. (Shanghai, China) in 100 mL of chitosan solution (Sigma Aldrich). The chitosan solution was made by adding approximately 1.5 g of chitosan (C6H11NO4)n, sourced from shrimp shells and containing ≥ 75% deacetylation, into 200 mL of a 2% acetic acid solution, followed by stirring continuously with a magnetic stirrer for 30 min. Next, ChNPs were formed by adding 100 mL of aqueous green tomato extract to the chitosan-TPP mixture (200 mL) dropwise, all while maintaining constant stirring for another 30 min. After this process, the supernatant was discarded through centrifugation at 10,000 g for 20 min. The resulting pellets were washed with distilled water and then freeze-dried using an ALPHA 1–2/LD-Plus vacuum to obtain nanoparticle powders.

## Characterization of Chitosan Nanoparticles

### Scan and Transmission Electron Microscope (SEM and TEM)

Transmission electron microscopy (TEM) (JEM-1230, JEOL, Akishima, Japan) was used to study the morphology of ChNPs. The procedure was followed by Abdallah et al. [38] and involved fixing the sample film in a grid box. Additionally, the morphology of ChNPs was also studied using scanning electron microscopy (SEM) (TM-1000, Hitachi, Tokyo, Japan) [38]. In short, a small amount of sample was fixed onto a carbon-coated copper grid to create a film. The SEM grid was exposed to a mercury lamp for five minutes in order to dry the film on it.

### Energy dispersive x-ray spectroscopy (EDX)

The samples were examined using an energy dispersive X-ray spectroscope (DX-700HS Shimadzu, Japan) to determine their elemental content. The equipment was attached to the SEM so that elemental information about the specimen being studied could be gathered.

### Determination of nanoparticle size

Dynamic Light Scattering (DLS) study using a Zetasizer nano (ZS) device (Malvern, UK) has been used to assess the particle size distribution and zeta potential of manufactured nanomaterials. After diluting the colloidal nanoparticle solution with 1 mL of distilled water, it was put in a disposable glass cuvette for examination [39].

### X-ray diffraction (XRD)

A common method for characterizing nanomaterials, X-ray diffraction (XRD) provides crucial phase identification data for manufactured nanoparticles. An X-ray diffractometer (X’ per PRO, Panalytical, The Netherlands) running with a Cu K radiation tube (= 1.54 A⸰) at 40 kV was used to confirm the physic-chemical crystalline character of the created nanomaterials. After that, the solution of colloidal nanoparticles was centrifuged at 16,000 × g for 30 min at 4 °C to produce a powder yield. The resulting nanoparticles were then allowed to air dry before being subjected to an X-ray for phase analysis [40].

### Fourier transform infrared spectroscopy (FTIR) analysis

The Fourier transform infrared spectrometer (Vector 22, Bruker, Bremen, Germany) was used to measure the dried powder of the bio-synthesized ChNPs at a range of 500–4000 cm^−1^ regions with resolution of 4 cm^−1^ to determine the functional group of ChNPs [238].

### Experiment design

The field experiment was conducted at the Experimental Farm of the Faculty of Agriculture, Ain Shams University, located in Shoubra El-Kheima, Qalyubia Governorate, Egypt (30.12° N, 31.25° E). Tomato seedlings were transplanted on October 22, 2023, and were maintained under standard agronomic practices until harvest. The experimental period lasted from October 2023 to January 2024. The soil at the experimental site was classified as clay loam, with a pH of 7.5, electrical conductivity (EC) of 1.9 dS m⁻^1^, organic matter content of 1.6%, and available nutrient levels of nitrogen (N), phosphorus (P), and potassium (K) at 48, 12, and 210 mg kg⁻^1^, respectively. Throughout the growing season, the average daily temperature ranged from 17.8 to 29.6 °C, while relative humidity varied between 55 and 68%. The average duration of sunshine was 9 h per day. There was no rainfall recorded during the experiment, and drip irrigation was utilized to maintain adequate soil moisture. These environmental conditions are typical of Egypt’s autumn–winter tomato growing season and were favorable for both tomato development and the progression of PVY disease. The field is organized into six plots, with each plot containing six rows. Each row measures 150 cm in length and 25 cm in width, with a distance of 25 cm between the rows. The field was irrigated and prepared before planting the seedlings. Tomato seedlings of the *S. lycopersicum* strain 010, aged 25 days, were planted and seven seedlings were planted in each row. The rows were grouped into eight groups, with each group consisting of three rows. Several preventive measures were implemented in the field to minimize the risk of unintentional infection by PVY or other viruses. The experimental site was isolated from other Solanaceae crops by a 2-m buffer zone planted with non-host vegetation. All seedlings were confirmed to be PVY-free through DAS-ELISA testing prior to transplantation. To reduce vector activity, yellow sticky traps and reflective mulches were installed, and an insecticide (imidacloprid at a concentration of 0.2 mL per liter) was applied weekly. Furthermore, volunteer and weed plants belonging to the Solanaceae family were removed throughout the growing season. These precautions helped maintain virus-free control plots, ensuring that any observed differences were solely due to the applied treatments.

- Group 1 (Ctrl): This is the control group, which was irrigated only with water throughout the experimental period. The seedlings in this group were neither infected with PVY nor treated with *B*. *subtilis* or ChNPs.
- Group 2 (PVY): The plants in this group were mechanically infected with the potato virus Y at the 5–6 leaf stage.
- Group 3 (Bs-only): Plants in this group were sprayed with a suspension of *B. subtilis* at a concentration of 1 × 10^–8^ cfu/ml.
- Group 4 (ChNPs-only): This group received a spray of chitosan nanoparticles solution at a concentration of 2 g/L.
- Group 5 (Bs + ChNPs): Plants in this group were sprayed with a mixture of chitosan nanoparticles solution and *B. subtilis* suspension in a 1:1 ratio.
- Group 6 (Pre-Bs): This group was treated with a foliar spray of *B. subtilis* suspension 48 h prior to being infected with the PVY virus.
- Group 7 (Pre-ChNPs): Plants in this group received a foliar spray of chitosan nanoparticles solution 48 h before virus infection.
- Group 8 (Pre-Combo): This group was treated with a foliar spray consisting of a mixture of ChNPs solution and *B. subtilis* suspension 48 h before infection with the PVY virus.

The plants in all groups were treated with foliar applications, which were administered twice at a two-day interval. Disease parameters were determined after 14 and 28 days of infection. In addition**,** measurements of morphological parameters and physiological analyses were conducted on the plants taken from the vegetative stage on December 11, 2023. The tomato fruit crop was harvested on January 30, 2024, for further measurements and physiological analyses of the fruits.

## Disease parameters

### Disease incidence (%)

Disease Incidence (%) was determined using the following formula:

$$=\frac{Number\;of\;infected\;plants}{Total\;number\;of\;plants}\times100$$

### Disease severity (%)

The following scale was used to assess the severity of the disease at 14 and 28 days after treatments: 0 indicates a plant with no symptoms, 1 indicates vein clearing symptoms on the leaves, 2 indicates mild mosaic symptoms on the leaves, 3 indicates moderate mosaic and curling of the leaves, and 4 indicates severe mosaic, deformation of the leaves, and notable stunting. The formula of Shalaby et al. [36] was used to determine the value of disease severity.

Determination of disease severity was calculated using the following equation:

$$DS\left(\%\right)=\sum\frac{Disease\;grade\times Number\;of\;plants\;in\;each\;grade}{Total\;number\;of\;plants\times Highest\;disease\;grade}\times100$$

### Determine the viral titer with DAS-ELISA

PVY poly-clonal antibody was utilized in DAS-ELISA to measure the titer of PVY from the control and treatment plants according to the method described by Clark and Adams [33].

### Determination of photosynthetic pigments

The approach outlined by Vernon et al. [41] was used to determine photosynthetic pigments. For five minutes, leaves with a known fresh weight were crushed in 85% aqueous acetone. Following centrifugation, 85% acetone was added to the supernatant until it reached a defined volume. With a spectrophotometer, the extraction was measured at three wave lengths (470, 649, and 665 nm) against a blank of pure 85% aqueous acetone. The following formulae were used to determine the amounts of chlorophyll a, b, and total chlorophyll in plant tissue:

- mg chlorophyll a/g tissue = 11.63 (λ 665) – 2.39 (λ649).
- mg chlorophyll b/g tissue = 20.11(λ649) – 5.18 (λ665).

Carotenoids were determined according to Lichtenthaler [42] equation:

- mg carotenoid/g tissue = (1000* λ 470) – (1.82 Chl a)- (85.02 Chl b)/198).

### Determination of malonaldehyde (MDA) content

To ascertain the amount of MDA, the thiobarbituric acid (TBA) assay was employed, following Heath and Packer [43] methodology. After 0.5 g of fresh samples were mixed in 5 ml of 0.1% trichloroacetic acid, they were centrifuged for 15 min at 10,000 × g. After heating the mixture at 95 °C for 30 min and rapidly cooling it in an ice bath, one milliliter of supernatant was combined with four milliliters of 0.5% thiobarbituric acid diluted in 20% TCA. The 532 nm absorbance was removed from the non-specific absorbance at 600 nm. Total MDA nmol/g fresh weight is the absorbance coefficient of malonaldehyde, which was determined using the extinction coefficient of 155 mM^–1^ cm^–1^.

### Determination of H2O2

The H_2_O_2_ level was measured in accordance with Zhou et al. [44]. Fresh plant tissue weighing a certain amount was mashed in phosphate buffer (50 mM, pH 6.5). For 20 min, the homogenate was centrifuged at 8000g. Phosphate buffer was added to the supernatant until it reached a specified volume (20 mL). 2.5 mL of 0.1% titanium sulfate in 20% sulphuric acid was combined with 0.5 mL of the supernatant in a dry, clean test tube, and the mixture was centrifuged at 8000 × g for 10 min. At 410 nm, the supernatant's absorbance was measured, and the absorbance results were compared to a standard curve created using known H_2_O_2_ concentrations.

### Determination of flavonoids

The method from Chen et al. [45] was used to determine total flavonoid content. A 0.5 g sample of fresh root, shoot, or fruit was extracted with 5 ml of 80% methanol and centrifuged at 10,000 × g for 10 min. The filtrate was adjusted to a known volume with methanol. For the analysis, 1 mL of the filtrate was mixed with 0.3 mL of 5% sodium nitrite and allowed to stand for 6 min. Then, 0.5 ml of 10% aluminum chloride was added, and after shaking, the mixture was neutralized with 2 mL of 1 M sodium hydroxide after another 6 min. After incubating for 10 min at room temperature, the absorbance was measured at 510 nm using a spectrophotometer. The total flavonoid content was calculated from a standard curve of quercetin and expressed as micrograms of quercetin per gram of fresh weight.

### Determination of total phenols

Total phenols were extracted and quantified using the method by Magalhães et al. [46]. A sample of 0.5 g of roots, shoots, and fruits was extracted with 40 mL of 80% cold methanol three times. The combined filtrates were diluted to a known volume with cold methanol. A 0.5 mL aliquot of the filtrate was mixed with 1 ml of 10% Folin-Ciocalteu reagent and allowed to stand for 3 min. Then, 1 mL of saturated sodium carbonate solution was added, and the mixture was shaken and left in the dark for 60 min. The optical density was measured at 725 nm using a spectrophotometer. Total phenol content was calculated based on a tannic acid standard curve and expressed as µg of tannic acid per gram of fresh weight.

### Determination of α-tocopherol activity

α-Tocopherol activity was measured according to Konyalιoğlu et al. [47]. Fresh tissue was homogenized in a 10 mL mixture of petroleum ether and ethanol (2:1.6 v/v) and centrifuged at 10,000 × g for 20 min. The supernatant was adjusted to a final volume of 50 ml with the solvent mixture. To 1 mL of the extract, 0.2 mL of 2% 2,2-dipyridyl in ethanol was added and mixed, then kept in the dark for 5 min. The red solution was diluted with 4 mL of distilled water, and a few drops of ferric chloride in ethanol were added. The color intensity of the aqueous layer was measured at 520 nm. α-Tocopherol content was calculated using a standard curve and expressed as µg/g of fresh weight.

### Determination of ascorbic acid (ASA)

Ascorbic acid was measured using the method by Mukherjee and Choudhuri [48]. A sample of 0.5 g from roots, shoots, or fruits was ground in 5 ml of 6% trichloroacetic acid (TCA) and centrifuged at 10,000 × g for 20 min. The filtrate was diluted to 10 mL with TCA. A 2 ml portion of the extract was mixed with 1 mL of 2% dinitrophenylhydrazine and one drop of 10% thiourea in 70% ethanol. This mixture was boiled for 15 min, cooled, and then treated with 2.5 mL of 80% sulfuric acid at 0°C. The absorbance was measured at 525 nm using a spectrophotometer, and ascorbic acid concentration was calculated from a standard curve.

### Determination of anthocyanin

Anthocyanins were extracted and quantified using Yan et al. [49] method, which involved adding 5 mL of acidic methanol (1% HCl) (v/w) to the known weight of fruits and shoots. After 18 h of incubation at 21 °C, the samples were centrifuged for three minutes at room temperature. The filtrate was diluted with 1% acidic methanol to a known volume. Using a spectrophotometer, the optical density at wave lengths of 530 and 657 nm was measured. The following formula was used to determine the amount of anthocyanins:

$$\begin{aligned}\mathrm Q\left(\mathrm{anthocyanins}\right)=&\left(\mathrm{Absorbance}\;\mathrm{at}\;530\;\mathrm{nm}\right.\\& \left.-0.25\;\mathrm{Absorbance}\;\mathrm{at}\;657\;\mathrm{nm}\right)\\&\times\mathrm{Fresh}\;\mathrm{weight}\left(\mathrm{in}\;\mathrm{grams}\right)\end{aligned}$$

## Determination of antioxidant enzymes

### Extraction of enzymes

A pestle and mortar were used for grinding four grams of fresh material in 0.05 M cold phosphate buffer (pH 6.5). The homogenate underwent a 10-min, 10,000 × g centrifugation. After using activated charcoal to adsorb the pigments, the supernatant was filtered. After the filtrate reached a predetermined volume, antioxidant enzymes were measured.

### Determination of peroxidase (EC 1.11. 1.7) enzyme

The oxidation of catechol by H_2_O_2_ was used to measure peroxidase activity using Tatiana et al. [50] method. Put 10–20 μL of enzyme extract, 2.5 mL of 50 mM sodium phosphate buffer (pH 6.1), 0.1 mL of 3% hydrogen peroxide, and 0.2 mL of 1% catechol in a clean, dry test tube. By adding 0.5 mL of 5% (v/v) H_2_SO_4_, the reaction was halted. The absorbance was measured with a spectrophotometer at 430 nm.

### Determination of polyphenol oxidase (EC. 1.14.18.1 or EC 1.10.3.2) enzyme

With minor adjustments, Meyer et al. [51] technique was used to measure polyphenol oxidase. Five milliliter of the assay mixture containing 125 µM of phosphate buffer pH 6.8, 100 µM catechol and 1.0 mL of crude enzyme extract was added in test tube. After five minutes of incubation at 25 °C, the reaction was stopped using 1.0 mL of 10% H_2_SO_4_. At 430 min, the color intensity was measured.

### Determination of catalase (EC 1.11.1.6) enzyme

The creation of a stable compound using ammonium molybdate was used by Singh et al. [52] to measure catalase activity. Place 0.2 mL of enzyme extract in a clean, dry test tube with 1.0 mL of reaction mixture that contained 65 mM H_2_O_2_ in 60 mM sodium phosphate buffer (pH 7.4). Following four minutes of incubation at 25 °C, 1.0 mL of 32.4 mM ammonium molybdate was added. A yellow complex of molybdate and H_2_O_2_ was produced, and the measurement was made using a spectrophotometer at 405 nm.

### Determination of amino acids

Total free amino acids were estimated using the method by Hwang and Ederer [53]. A half gram of plant tissue was homogenized in 10 mL of 70% boiling ethanol and then filtered. The residue was re-extracted with 70% ethanol, and the combined filtrate was brought to a final volume of 50 mL. In a clean test tube, 1 mL of the filtrate was mixed with 1 mL of ninhydrin reagent, covered with aluminum foil, and heated in a boiling water bath for 12 min. After cooling for 15 min, the optical density was measured at 580 nm using a spectrophotometer. The total free amino acids were calculated from a standard graph prepared with glycine, expressed as mg of glycine per gram of fresh weight.

### Determination of Proline

The colorimetric approach, as outlined by Bates et al. [54], was used to determine proline. Five milliliters of 3% aqueous sulfosalicylic acid were used to homogenize a specified weight of plant material, and the resulting homogenate was then filtered through Whatman filter paper. Two milliliters of filtrate, two milliliters of acid ninhydrin, and two milliliters of glacial acetic acid were heated in a test tube for one hour at 100 °C in a boiling water bath. The reaction was then stopped in an ice bath. Four milliliters of toluene were used to extract the reaction mixture, which was then aggressively stirred for fifteen to twenty seconds. Using toluene as a blank test, the absorbance was measured at 520 nm after the chromophore containing toluene was aspirated from the aqueous phase and warmed to room temperature. A proline standard curve was used to calculate the proline concentration.

### Determination of total soluble sugar

The phenol sulfuric technique, as described by Dubois et al. [55], was used to calculate the total soluble sugars. After grinding the fresh material in 70% ethanol, the ethanolic extract was extracted using a centrifuge. Five milliliters of strong sulfuric acid and 0.5 mL of 5% phenol were added to a test tube containing one milliliter of ethanolic extract, and everything was carefully mixed. After 30 min of the tubes being left at room temperature, a spectrophotometer was used to measure the optical density of the solutions at 490 nm.

### Determination of total soluble protein

A half gram of powdered roots and shoots were crushed in 5 mL of phosphate buffer, it was centrifuged for 10 min at 6000 × g. While the residue was being cleaned with two milliliters of distilled water, the supernatant separated in a test tube. To determine the total soluble proteins, the supernatant and washing were mixed. Folin-Cicalteu reagent is used to measure the total soluble protein level. Samples' optical densities were measured at 750 nm [56].

### Determination of lycopene

The Nagata and Yamashita [57] methodology was used to measure the amount of lycopene in tomato fruits. Specifically, freeze-dried fruits were ground into a powder, and 100 mg of that powder were combined with a 10-ml solution that contained two parts acetone and three parts hexanes. After agitating the resultant solution vigorously for ten minutes, a Whatman number 1 filter paper was used to filter it. The filtrate's absorbance was measured at 453, 505, 645, and 663 nm. The three tests' average mean values were expressed as milligrams of lycopene per milliliter of FW. Three duplicates of each test were performed. The following formulas were used to calculate the lycopene contents:

$$\begin{aligned}Lycopene\left(mg/100\;mL\right)=&\left(0.0458\times A663\;nm\right)+\left(0.204\;\ast A645\;nm\right)\\&+\left(0.372\times A505\;nm\right)\_\left(0.0806\times A453\;nm\right)\end{aligned}$$

### Determination of 2,2-diphenyl-1-picrylhydrazyl (DPPH)

About 0.25 g of tomato fruit was weighed, and then 1 mL of DPPH was added. The final volume is precisely 5 mL with the addition of ethanol. Thirty minutes are given for the mixture to stand. For 20 s, homogenization is done. The absorption was measured using a spectrophotometer at optical density 580 nm. Ethanol-based tomato juice solution. The control solution is DPPH without the test solution added [58].

### Statistical analysis

One-way analysis of variance (ANOVA) was performed on all experimental data, and Duncan's multiple range test was used to identify significant differences between treatment means at p < 0.05. The mean ± standard deviation from a minimum of three separate biological replicates is represented for each value. The normalization and structuring of the data for multivariate studies, such as principal component analysis (PCA) and hierarchical clustering. (Addinsoft, New York, USA) XLSTAT software, version 2024.1.1, was used for all statistical and chemometric studies [59, 60].

## Results

### Viral isolation, confirmation and molecular characterization

**Fig. 1:**
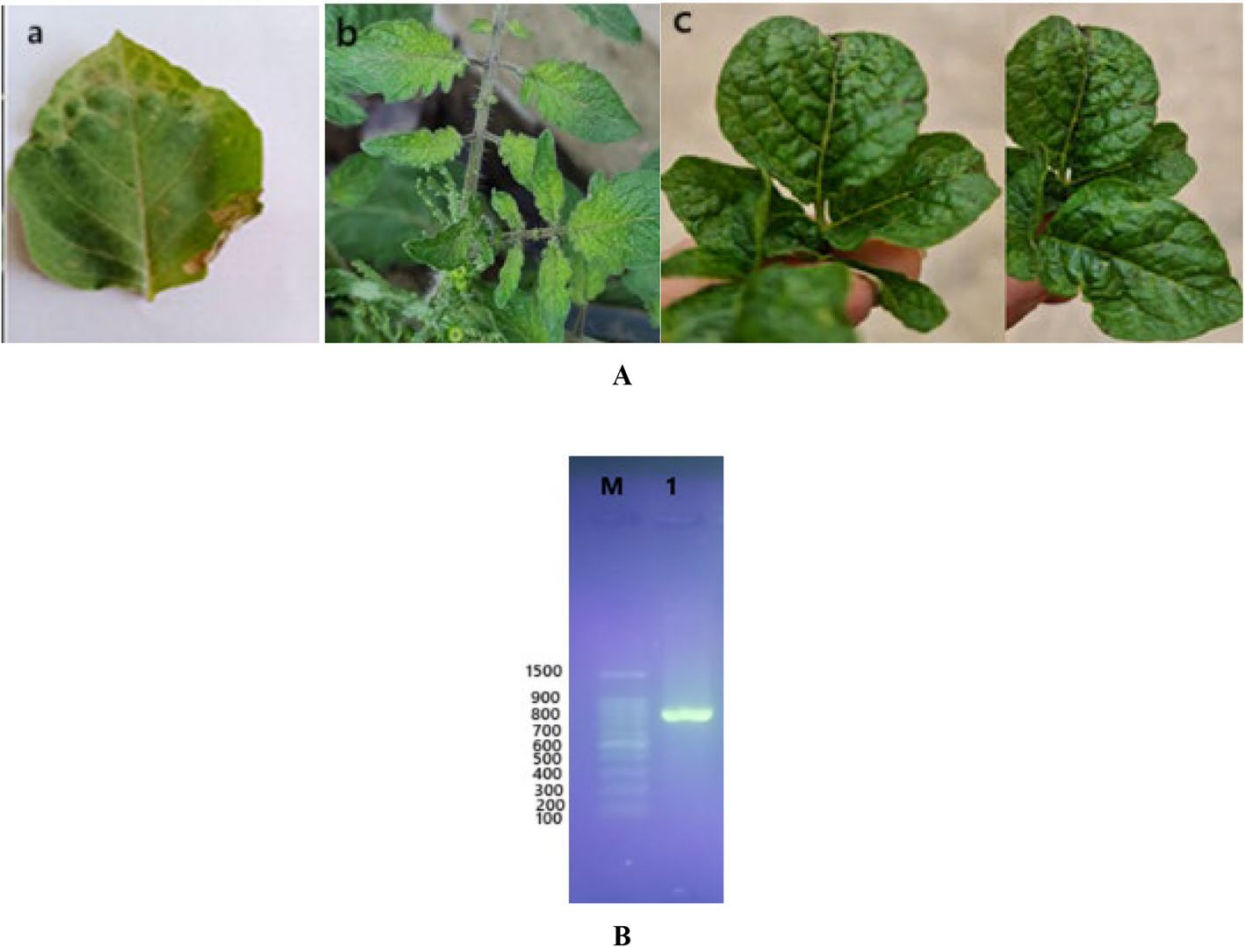
photograph of plants mechanically inoculated with PVY isolate showing different symptoms ( **A**) a: vein clearing, mottling, sever mosaic on *D. metel*; b: mottling and mosaic on *S. lycopersicum*; c: mosaic, necrosis, deformation and leaf narrow on *Solanum tuberosum* within 7 to 14 days under controlled greenhouse conditions. **B** Detection of Potato virus Y (PVY) by RT-PCR and 1%agarose gel electrophoresis analysis. Lane M, hundred ladder marker, Lan1 1, PVY coat protein gene amplicon

In mechanical inoculation tests, the sap extracted from symptomatic tomato leaves induced typical PVY-associated symptoms on the 3 PVY inoculated plants (*Datura metel, Solanum lycopersicum and Solanum tuberosum*). The obtained results illustrated that *Datura metel* showed vein clearing, mottling, sever mosaic and leaf deformation while *Solanum lycopersicum* showed mottling and mosaic and *Solanum tuberosum* showed mosaic, necrosis, deformation and leaf narrow within 7 to 14 days under controlled greenhouse conditions (Fig. 1A).

### Confirmation the PVY with serology

The symptomatic tomato leaf samples were tested for Potato virus Y (PVY) using DAS-ELISA, and recorded positive results 1.5 O. D with compared to negative control that recorded 0.089 O.D.

### Molecular confirmation with RT-PCR

In the present study, RT-PCR was performed on the extracted total RNA from infected plant tissues with specific primers of PVY coat protein (CP) gene. Analysis of PCR products in agarose gel electrophoresis revealed that the amplification of a specific band was approximately 801 bp as shown in Fig. 1B.

### Characterization of nanoparticles

**Fig. 2:**
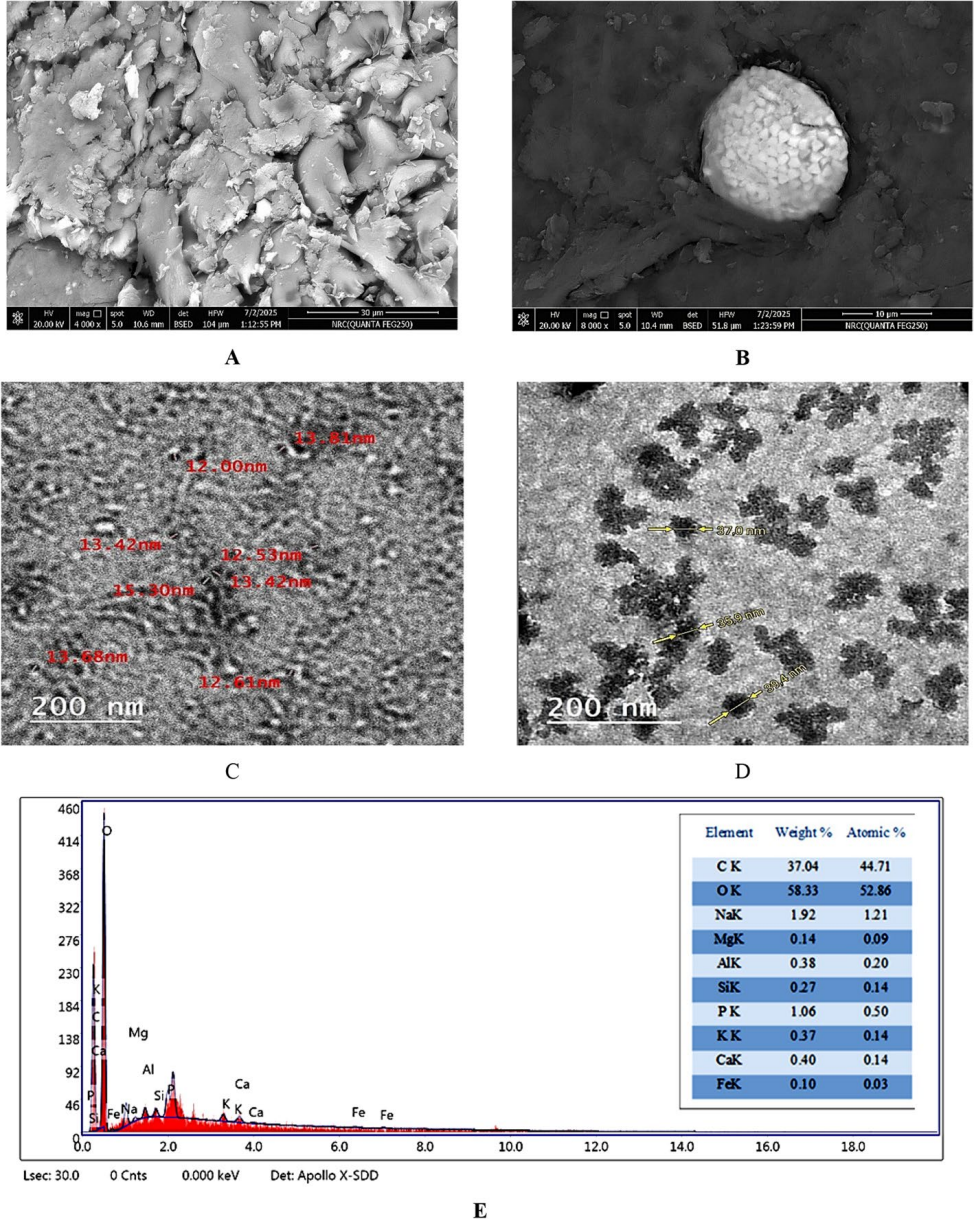
Characterization of ChNPs determined by SEM (**A**, **B**), TEM (**C**, **D**) and EDX (**E**)

The data in Fig. 2A-D depicts an investigation of the morphology of biosynthesized chitosan nanoparticles using SEM and TEM, respectively. The morphology of all nanoparticles was relatively homogeneous, with a quite consistent particle size distribution and spherical in shape. The SEM analysis indicates spherical particles with a smooth surface and particles in the agglomerated state. While, TEM analysis of the obtained biosynthesized chitosan nanoparticles reveals particles ranging in size between 12.0 and 39.4 nm, the particles are spherical.

The elemental composition of the biosynthesized ChNPs was confirmed by using EDX analysis. The EDX spectra revealed the percentage elemental composition of ChNPs, composed of carbon (37.04%), oxygen (58.33%), sodium (1.92%), phosphorus (1.06%), and other elements such as Mg, Al, Si, K, Ca and Fe in minor proportion (Fig. 2E).

**Fig. 3:**
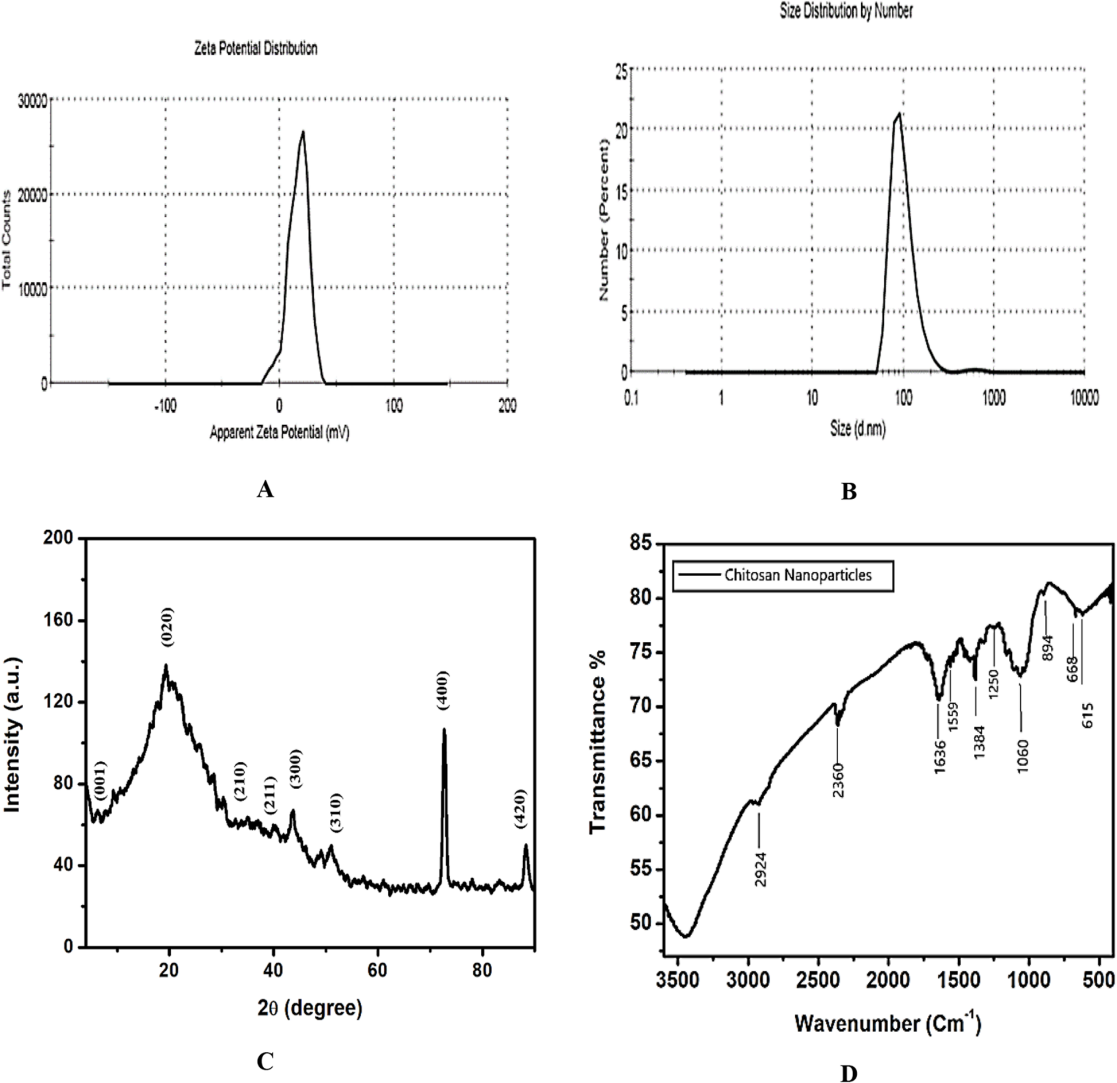
Characterization of ChNPs determined by the ζ-potential (**A**), particles size analysis (**B**), XRD (**C**), and FTIR (**D**)

To determine the stability of the nanoparticles, the Z potential was measured. The chitosan nanoparticles prepared using the nanoprecipitation method had a + 36.5 mV Z potential (Fig. 3A). Analysis of the size and homogeneity of nanoparticles was determined using the DLS method. Based on the results of measurements, the size of ChNPs obtained was 198.2 nm as shown in Fig. 3B.

The X-ray diffraction (XRD) pattern of the biosynthesized chitosan nanoparticles (Fig. 3c) displayed characteristic diffraction peaks at approximately 2θ values of 3.17°, 19.13°, 28.7°, 30.4°, 43.7°, 49.2°, 72.6°, and 88.3°. These peaks correspond to the (001), (020), (210), (211), (300), (310), (400), and (420) planes, respectively. The prominent peak around 19° indicates the semi-crystalline nature of chitosan, while the additional minor peaks suggest a partial ordering within the polymeric matrix or the presence of bioactive compounds derived from the fruit extract used during the synthesis.

FT–IR analysis was conducted for characterization and identification of the functional groups found in the biosynthesized ChNPs (Fig. 3D). A peak at 2924 to 2360 cm^−1^ was observed for the main functional group of chitosan and is due to the O–H group of stretching vibrations. The presence of absorption peaks at 1636, 1559, and 1384 cm^−1^ are due to the N–H bending vibration of protonated amino (-NH_2_) group and C-H bending vibration of the alkyl group. The absorption peaks at 1250, 1060 and 894 cm^−1^ are recognized due to the anti-symmetric stretching vibration of C–O–C bridges and assigned to glucopyranose ring in chitosan matrix. The presence of absorption peaks at 668 and 615 cm^−1^ are due to C-O stretching vibrations within the chitosan molecule.

### Changes on viral infectivity and disease severity

The protective antiviral activity of *B. subtilis*, ChNPs, and their combination against PVY was evaluated. The results show that foliar spraying of Bs and ChNPs and their combination substantially reduced the virus concentration, the percentage of infection with the virus, and the disease severity (DS), compared to challenge control.

**Fig. 4:**
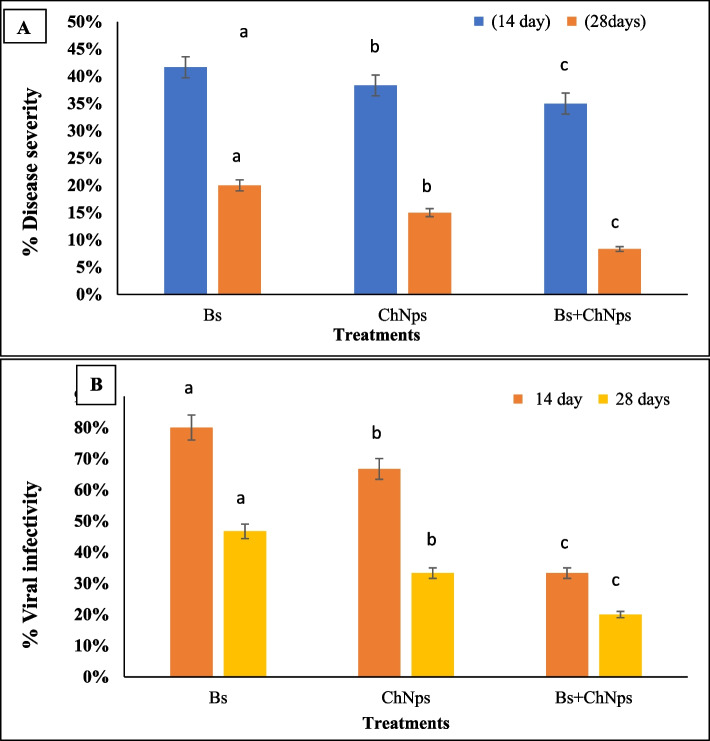
Effect of ChNPs, *B. subtilis* and combination on the disease severity (**A**), and infectivity (**B**) in tomato after 14 and 28 days of pre-inoculation. The average values (SD) for each treatment were determined from three replications. Means that are labeled with different letters in each bar indicate significant differences based on Duncan's Multiple Range Test at *p* < 0.05

The data obtained demonstrates a clear antiviral effect of *B. subtilis*, chitosan nanoparticles (NPS), and their combination in reducing disease severity caused by Potato virus Y (PVY) in tomato plants. Fourteen days post-inoculation, the percentage of disease severity was highest in plants treated with *B. subtilis* alone (41.67%), followed by chitosan NPs (38.33%), while the combined treatment of *B. subtilis* and chitosan NPs showed the most effective protection, reducing disease severity to 35.00%. This protective trend became more pronounced after 28 days, with disease severity dropping to 20% for *B. subtilis*, 15% for chitosan NPs, and only 8.33% for the combined treatment. These results indicate that while both *B. subtilis* and chitosan NPs exhibit antiviral activity individually, their combination has a synergistic effect, offering enhanced protection against PVY in tomatoes over time as shown in Fig. 4A.

In terms of viral infectivity, a similar trend was observed. At 14 days, viral infectivity levels were recorded at 80% for *B. subtilis*, 66.70% for ChNPs, and significantly lower at 33.30% for the combined treatment. By 28 days, infectivity was further reduced to 46.70% for *B. subtilis*, 33.30% for ChNPs, and the lowest level of 20% for the combination. These findings suggest that both *B. subtilis* and chitosan NPs possess antiviral activity individually; however, their combined application substantially enhances protection by reducing both disease severity and viral infectivity more effectively than either treatment alone (Fig. 4B). Overall, the combined application of *Bacillus subtilis* and chitosan nanoparticles proved most effective, especially when applied before infection, significantly reducing both disease severity and viral infectivity of PVY in tomato plants.

### Changes on virus accumulation content

All treatments (ChNPs, *B. subtilis*, and combination) appreciably decreased virus accumulation in treated tomato plants. All treatments (ChNPs, *B. subtilis*, and combination) significantly reduced virus accumulation in the treated plants when applied before inoculation with PVY (Table 1). The highest activity was obtained when the plants were sprayed with combination between ChNPs and *B. subtilis* compared to the positive control inoculated with PVY. A significant difference was recorded after treatment with combination between ChNPs and *B. subtilis*, followed by *B. subtilis* and ChNPs with the virus content significantly decreased by 89.56% (0.26 OD), 89.56% (0.26 OD), and 86.75% (0.33 OD), respectively, compared to the untreated positive control.

**Table 1:** Effect of ChNPs, *B. subtilis* and combination on virus concentration and accumulation contents using ELISA reaction

Treatment	O.D after 30 min	%	O.D after 45 min	%	O.D after 60 min	%
PVY	1.40 ± 0.2^a^	-	2.17 ± 0.3^a^		2.49 ± 0.3^a^	
PVY + *B*. *subtills*	0.20 ± 0.02^b^	−85.71	0.25 ± 0.03^b^	−88.48	0.26 ± 0.01^b^	−89.56
PVY + ChNps	0.21 ± 0.01^b^	−85.00	0.30 ± 0.02^b^	−86.18	0.33 ± 0.02^b^	−86.75
PVY + Combo	0.20 ± 0.01^b^	−85.71	0.26 ± 0.02^b^	−88.02	0.26 ± 0.02^b^	−89.56

The average values (SD) for each treatment were determined from three replications. Means that are labeled with different letters in each bar indicate significant differences based on Duncan's Multiple Range Test at *p* < 0.05

### Changes in vegetative growth traits

Vegetative growth traits exhibited marked differences across the treatment groups, highlighting the impact of PVY infection and the varying efficacy of biostimulant applications and priming strategies (Table 2). The uninfected control group provided a physiological baseline, with shoot length (40.2 cm), root length (22.4 cm), and plant height (73.2 cm) reflecting average growth under unstressed conditions. In contrast, plants infected with PVY showed a dramatic reduction at *p* < 0.05 in growth, with shoot length dropping (39.8%), root length (25.9%), shoot fresh weight (72.4%), root fresh weight (57.1%), shoot dry weight (76.2%) and root dry weight (54.8%). This decline was accompanied by significant reductions in leaf number (37.1%), leaf area (77.4%), and plant height (24.3%), aligning with the well-documented stunting and leaf degeneration typically induced by viral stress.

**Table 2:** Effect of ChNPs, *B. subtilis* and combination on vegetative growth traits of tomato plants under PVY infection

Treatments	Shoot length (cm)	Root length (cm)	Plant height (cm)	Shoot fresh weight (g)	Root fresh weight (g)	Shoot dry weight (g)	Root dry weight (g)	Number of leaves/plant	Leaves area/plant cm^2^
Ctrl	40.2 ± 6.02^a^	22.4 ± 2.19^c^	73.2 ± 4.97^ab^	152.4 ± 18.06^ cd^	9.92 ± 0.29^ cd^	18.24 ± 0.78^e^	2.08 ± 0.16^c^	17.8 ± 5.07^ cd^	1470.0 ± 228.58^d^
PVY	24.2 ± 3.11^e^	16.6 ± 1.95^e^	55.4 ± 10.64^c^	42.0 ± 3.74^e^	4.26 ± 0.25^e^	4.34 ± 0.42^ g^	0.94 ± 0.11^e^	11.2 ± 0.84^d^	332.0 ± 44.38^f^
Bs-only	52.4 ± 6.02^c^	32.0 ± 2.0^ cd^	78.6 ± 15.27^b^	357.4 ± 56.60^ cd^	11.42 ± 0.43^d^	38.86 ± 0.74^d^	3.28 ± 0.47^c^	24.0 ± 7.00^bc^	4364.0 ± 338.28^e^
ChNPs-only	60.0 ± 8.97^b^	35.4 ± 3.36^b^	79.4 ± 15.14^ab^	152.6 ± 8.23^b^	10.2 ± 0.57^b^	15.04 ± 0.69^b^	2.12 ± 0.28^b^	16.4 ± 2.70^b^	1986.0 ± 170.38^b^
Bs + ChNPs	64.0 ± 6.63^a^	38.2 ± 6.18^a^	89.0 ± 9.77^a^	556.0 ± 196.08^a^	14.38 ± 0.88^a^	66.6 ± 1.14^a^	6.02 ± 0.29^a^	31.6 ± 7.30^a^	8102.0 ± 583.54^a^
Pre-Bs	32.2 ± 2.28^d^	18.6 ± 2.61^de^	54.6 ± 7.40^c^	101.6 ± 16.35^cde^	10.2 ± 1.48^ cd^	12.02 ± 0.33^f^	1.06 ± 0.30^e^	14.4 ± 4.04^ cd^	1192.0 ± 128.72^e^
Pre-ChNPs	37.6 ± 2.30^ cd^	21.8 ± 1.79^ cd^	49.8 ± 3.70^d^	92.0 ± 2.55^de^	10.540 ± 0.75^bcd^	14.38 ± 0.48^e^	1.5 ± 0.31^d^	15.4 ± 2.41^ cd^	2216.0 ± 259.48^ cd^
Pre-Combo	47.2 ± 2.86^b^	24.4 ± 3.97^c^	72.4 ± 11.65^b^	197.4 ± 16.43^c^	11.2 ± 0.57^bc^	28.76 ± 0.51^c^	2.14 ± 0.25^c^	23.4 ± 3.36^b^	2550.0 ± 103.44^c^

The average values (SD) for each treatment were determined from three replications. Means that are labeled with different letters in each bar indicate significant differences based on Duncan's Multiple Range Test at *p* < 0.05

Among the healthy plants treated with biostimulants, the combined application of *B. subtilis* and chitosan nanoparticles led to the most pronounced enhancement in vegetative parameters. This group exceeded control values in shoot length (59.2%), root length (70.5%), leaf area (451.2%), plant height (21.6%), shoot fresh weight (264.8%), root fresh weight (45.0%), shoot dry weight (265.1%) and root dry weight (189.4%) indicating a strong growth-promoting effect in the absence of infection. Chitosan nanoparticles alone also supported robust development, with shoot and root lengths reaching 49.3% and 58.0%, respectively. However, treatment with *Bacillus subtilis* alone resulted in more modest improvements (shoot:30.3%, root: 42.9%), suggesting limited effectiveness when applied independently under non-stressed conditions.

Primed plants, those pretreated with biostimulants before viral inoculation exhibited varied levels of physiological resilience. The combination priming strategy demonstrated the best protective effect, increasing shoot and root lengths by about 95.0% and 47.0%, leaf area by about 668.1%, plant height (30.7%), shoot fresh weight (370.0%), root fresh weight (162.9%), shoot dry weight (562.7%), and root dry weight (127.7%). These values were significantly higher than those of the PVY-infected group (*p* < 0.05) and closely approached those of the control group. Priming with *Bacillus subtilis* or chitosan nanoparticles alone yielded less consistent outcomes.

### Changes in photosynthetic pigments

Photosynthetic pigment content varied notably across treatments, highlighting the effects of PVY infection and biostimulant application on pigment retention (Table 3). In the control group, moderate levels of chlorophyll a (1.49 mg/g FW), chlorophyll b (0.90 mg/g FW), and total chlorophyll (2.39 mg/g FW) were observed. The chlorophyll a/b ratio was 1.65, reflecting a balanced pigment composition under non-stressed conditions.

**Table 3:** Effect of ChNPs, *B. subtilis* and combination on photosynthetic pigments of tomato plants under PVY infection

Treatments	Chl. a (mg/g FW)	Chl. b (mg/g FW)	Chlorophyll a/b Ratio	Total Chl	Carotenoid (mg/g FW)	Carotenoid/Total Chlorophyll	Total pigments (mg/g FW)
Ctrl	1.489 ± 0.05^ab^	0.901 ± 0.01^c^	1.654 ± 0.06^bc^	2.390 ± 0.05^c^	1.638 ± 0.11^b^	0.686 ± 0.06^a^	4.028 ± 0.10^c^
PVY	0.449 ± 0.02^d^	0.227 ± 0.04^e^	2.012 ± 0.34^ab^	0.676 ± 0.04^e^	0.343 ± 0.01^e^	0.509 ± 0.04^b^	1.019 ± 0.04^f^
Bs-only	1.146 ± 0.17^c^	0.893 ± 0.15^c^	1.332 ± 0.45^bc^	2.038 ± 0.05^d^	1.592 ± 0.10^bc^	0.782 ± 0.06^a^	3.631 ± 0.09^d^
ChNPs-only	1.686 ± 0.13^a^	1.129 ± 0.08^b^	1.493 ± 0.04^bc^	2.815 ± 0.21^b^	1.978 ± 0.13^a^	0.705 ± 0.07^a^	4.793 ± 0.25^b^
Bs + ChNPs	1.754 ± 0.01^a^	1.426 ± 0.01^a^	1.230 ± 0.02^c^	3.179 ± 0.01^a^	2.103 ± 0.09^a^	0.662 ± 0.03^a^	5.283 ± 0.08^a^
Pre-Bs	1.321 ± 0.07^bc^	0.541 ± 0.02^d^	2.443 ± 0.17^a^	1.862 ± 0.07^d^	0.856 ± 0.08^d^	0.461 ± 0.06^b^	2.718 ± 0.06^e^
Pre-ChNPs	1.309 ± 0.36^bc^	0.877 ± 0.11^c^	1.531 ± 0.59^bc^	2.186 ± 0.30^ cd^	1.408 ± 0.25^c^	0.654 ± 0.15^a^	3.594 ± 0.35^d^
Pre-Combo	1.324 ± 0.17^bc^	0.780 ± 0.24^c^	1.829 ± 0.68^abc^	2.104 ± 0.32^ cd^	1.648 ± 0.04^b^	0.794 ± 0.12^a^	3.752 ± 0.33^ cd^

The average values (SD) for each treatment were determined from three replications. Means that are labeled with different letters in each bar indicate significant differences based on Duncan's Multiple Range Test at *p* < 0.05

PVY infection caused severe pigment degradation (*p* < 0.05). Total chlorophyll dropped by about 71.7%, with chlorophyll a and b falling about 69.9% and 47.8%, respectively, the lowest values across all treatments. This was accompanied by a relatively high chlorophyll a/b ratio (21.6%), indicating disproportionate loss of chlorophyll b and a possible compensatory stress response. Carotenoid levels were also significantly reduced about 79.1%, and total pigment content declined about 74.7%.

Among the healthy plants treated with biostimulants, the combination of *Bacillus subtilis* and chitosan nanoparticles led to the highest pigment accumulation (*p* < 0.05). Total chlorophyll reached increased about 33.0%, and carotenoid content peaked about 28.4%. The total pigment concentration significantly exceeded that of the control about 31.2%, confirming a strong photosynthetic enhancement effect. Chitosan nanoparticles alone also maintained high chlorophyll and carotenoid levels, while *Bacillus subtilis* alone was less effective.

Priming before infection produced varied outcomes. The group primed with both agents showed pigment recovery, with increasing in total chlorophyll by about 211.0% and total pigments 268.4% above the infected plants. In contrast, *Bacillus*-primed and chitosan nanoparticles plants exhibited significantly increased in all the above parameters as compared to infected plants with PVY virus.

### Changes in oxidative stress markers

**Fig. 5:**
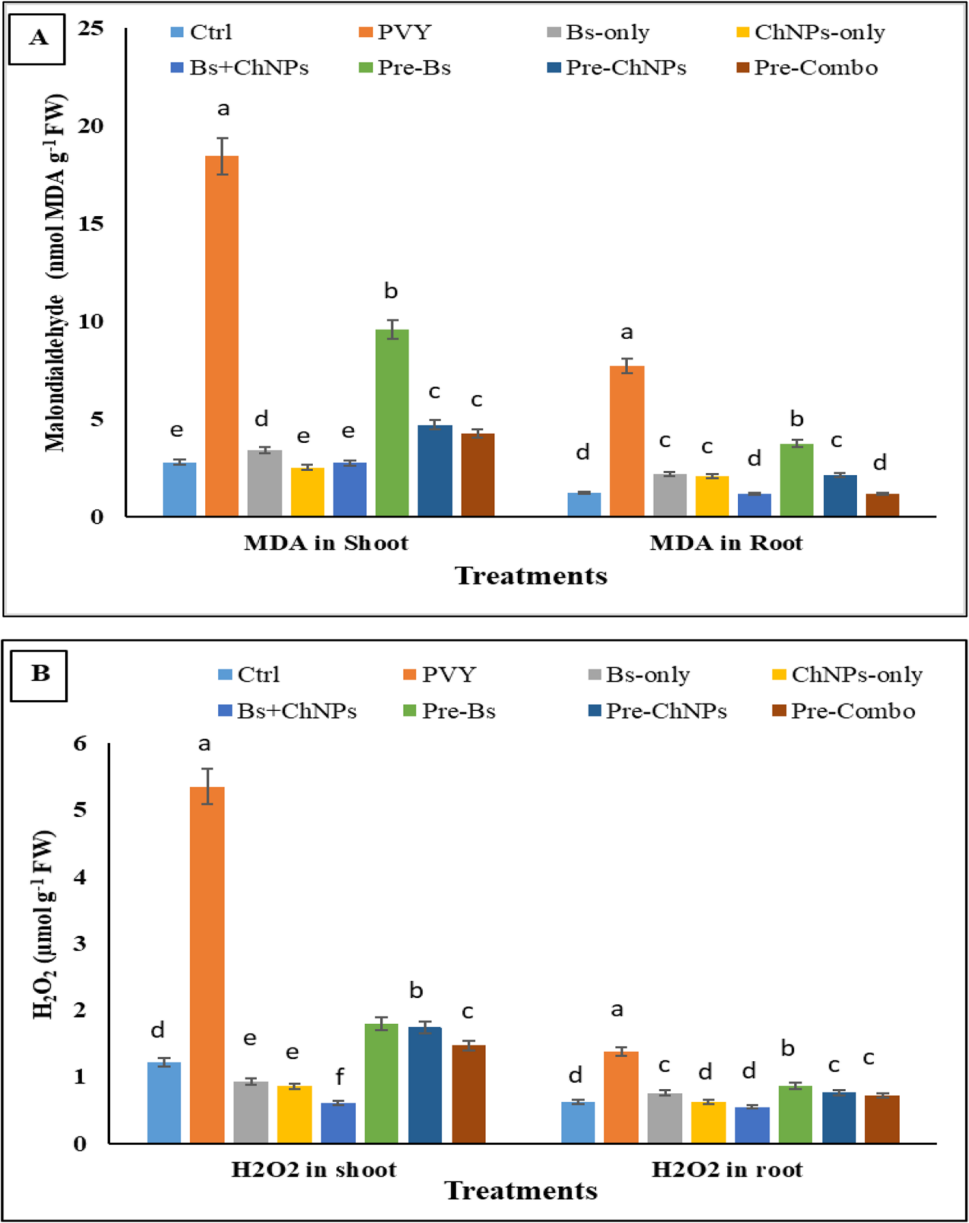
Effect of ChNPs, *B. subtilis* and combination on MDA (**A**), and H_2_O_2_ (**B**) in shoots and roots of tomato. The average values (SD) for each treatment were determined from three replications. Means that are labeled with different letters in each bar indicate significant differences based on Duncan's Multiple Range Test at *p* < 0.05

Oxidative stress indicators varied significantly across treatments (*p* < 0.05), reflecting the extent of cellular damage under PVY infection and the effectiveness of mitigation strategies (Fig. 5A, B). In the control group, baseline levels of malondialdehyde (MDA) were 2.70 nmol/g FW in shoots and 1.18 nmol/g FW in roots, while H₂O₂ levels measured 1.21 and 0.62 µmol/g FW, respectively, indicative of stable redox balance in healthy plants. PVY infection triggered a sharp rise in oxidative markers. Shoot MDA spiked about 583.7%, and H₂O₂ about 341.2%, with root values also markedly elevated by about 558.7%, and 120.5%, respectively. These values represent the highest across all treatments and confirm the destructive impact of viral stress on membrane integrity and ROS accumulation.

Application of biostimulants to uninfected and infected plants significantly reduced oxidative markers. The combined treatment with *B. subtilis* and chitosan nanoparticles lowered shoot MDA by about 1.0% and H₂O₂ about 49.7% matching or decreasing upon control values. Priming treatments offered partial protection. The Pre-Combo group had lower oxidative stress than the PVY group, with shoot and root MDA about 76.8%, 84.4% and H₂O₂ about 72.6%, and 47.7%, respectively as compared to infected plants.

### Changes in non-enzymatic antioxidants

Non-enzymatic antioxidant levels exhibited significant variation among treatments (*p* < 0.05), reflecting the differential capacities of treatments to enhance or restore antioxidant pools in tomato tissues (Table 4). In the control group, baseline levels of flavonoids (2.73 µg/g), total phenols (2.36 µg/g), α-tocopherol (0.72 µg/g), and ascorbic acid (7.66 µg/g) in shoots were notably low, as were root levels of flavonoids (0.74 µg/g), total phenols (1.25 µg/g), α-tocopherol (0.51 µg/g), and ascorbic acid (0.31 µg/g).

**Table 4:** Effect of ChNPs, *B. subtilis* and combination on non-enzymatic antioxidants in shoots and roots of tomato plants under PVY infection

Treatments	Flavonoids in shoot (µg quercetin/g)	Flavonoids in root (µg quercetin/g)	Total phenol in shoot (µg tannic acid/g)	Total phenol in root (µg tannic acid/g)	α-tochopherol in shoot (µg/g FW)	α-tochopherol in root (µg/g FW)	Ascorbic acid in shoot (µg/g FW)	Ascorbic acid in root (µg/g FW)	Anthocyanin in shoot (g/ml FW)
Ctrl	2.733 ± 0.50^ h^	0.742 ± 0.04^ g^	2.362 ± 0.03^ h^	1.247 ± 0.37^d^	0.722 ± 0.10^f^	0.510 ± 0.02^e^	7.661 ± 0.20^ g^	0.313 ± 0.01^d^	1.400 ± 0.10^f^
PVY	8.300 ± 0.50^ g^	1.858 ± 0.15^f^	5.488 ± 0.02^ g^	2.600 ± 0.25^c^	2.516 ± 0.25^e^	0.856 ± 0.71^de^	15.699 ± 0.1^e^	0.808 ± 0^c^	2.482 ± 0.07^e^
Bs-only	14.750 ± 0.00 e	2.708 ± 0.10^d^	7.563 ± 0.02^d^	3.357 ± 0.31^bc^	3.233 ± 0.32^d^	1.277 ± 0.05^ cd^	11.448 ± 0.1^f^	0.937 ± 0.01^c^	5.775 ± 0.07^b^
ChNPs-only	18.342 ± 0.39^b^	3.392 ± 0.08^c^	9.092 ± 0.21^b^	4.123 ± 0.55^b^	4.971 ± 0.30^a^	2.116 ± 0.19^ab^	17.861 ± 0.2^c^	1.131 ± 0.05^c^	6.956 ± 0.23^a^
Bs + ChNPs	22.942 ± 0.19^a^	4.600 ± 0.48^a^	11.159 ± 0.19^a^	5.783 ± 0.45^a^	5.284 ± 0.51^a^	2.466 ± 0.10^a^	19.640 ± 0.5^b^	1.185 ± 0.1^c^	6.367 ± 0.26^ab^
Pre-Bs	13.408 ± 0.10^f^	2.366 ± 0.10^e^	6.254 ± 0.17^f^	3.077 ± 1.27^bc^	4.000 ± 0.11^c^	1.616 ± 0.19^bc^	16.731 ± 0.0^d^	1.058 ± 0.03^c^	3.267 ± 1.01^d^
Pre-ChNPs	15.442 ± 0.06^d^	3.183 ± 0.05^c^	6.645 ± 0.17^e^	3.230 ± 0.67^bc^	4.455 ± 0.07^bc^	1.639 ± 0.15^bc^	17.320 ± 0.7^ cd^	1.739 ± 0.57^b^	3.902 ± 0.13^ cd^
Pre-Combo	17.625 ± 0.28^c^	4.125 ± 0.04^b^	8.746 ± 0.13^c^	5.653 ± 0.21^a^	4.834 ± 0.26^ab^	1.793 ± 0.24^bc^	20.429 ± 0.3^a^	3.407 ± 0.01^a^	4.252 ± 0.01^c^

The average values (SD) for each treatment were determined from three replications. Means that are labeled with different letters in each bar indicate significant differences based on Duncan's Multiple Range Test at *p* < 0.05

PVY infection caused a moderate but significant rise in all antioxidants compared to the control, particularly in shoot tissues, where total flavonoids and phenols increased by about 203.7% and132.3%, respectively, α-tocopherol, ascorbic acid and anthocyanin rose about 248.5%, 104.9% and 77.3%, respectively above control values. However, these values remained below those observed in most treatment groups, suggesting only a limited stress-induced response.

Biostimulants applied to uninfected plants markedly enhanced non enzymatic antioxidant accumulation. The combined application of *B. subtilis* and chitosan nanoparticles led to the highest shoot values for flavonoids (739.4%), total phenols (372.4%), α-tocopherol (631.9%), ascorbic acid (156.4%) and anthocyanin (354.8%), while also achieving the highest root concentrations across all parameters. Chitosan alone also performed strongly, especially in shoots, but was less consistent in roots. Conversely, *Bacillus* alone showed a relatively lower capacity to enhance antioxidant pools, particularly in root tissues.

Priming prior to infection produced more variable results. The dual-agent priming strategy performed best, significantly improving both shoot and root non enzymatic antioxidant content. Flavonoids, phenols, α-tocopherol, ascorbic acid, and anthocyanin in shoot increased about 112.2%, 59.4%, 92.1%, 30.1%, and 71.3%, respectively, while in roots peaked by about 122.0%, 117.4%, 109.5%, and 321.7%, respectively higher than infected plants. Pre-treatment with either *Bacillus* or chitosan alone resulted in intermediate improvements but did not fully restore antioxidant levels to the range seen in combined treatments.

### Changes in enzymatic antioxidants

**Fig. 6:**
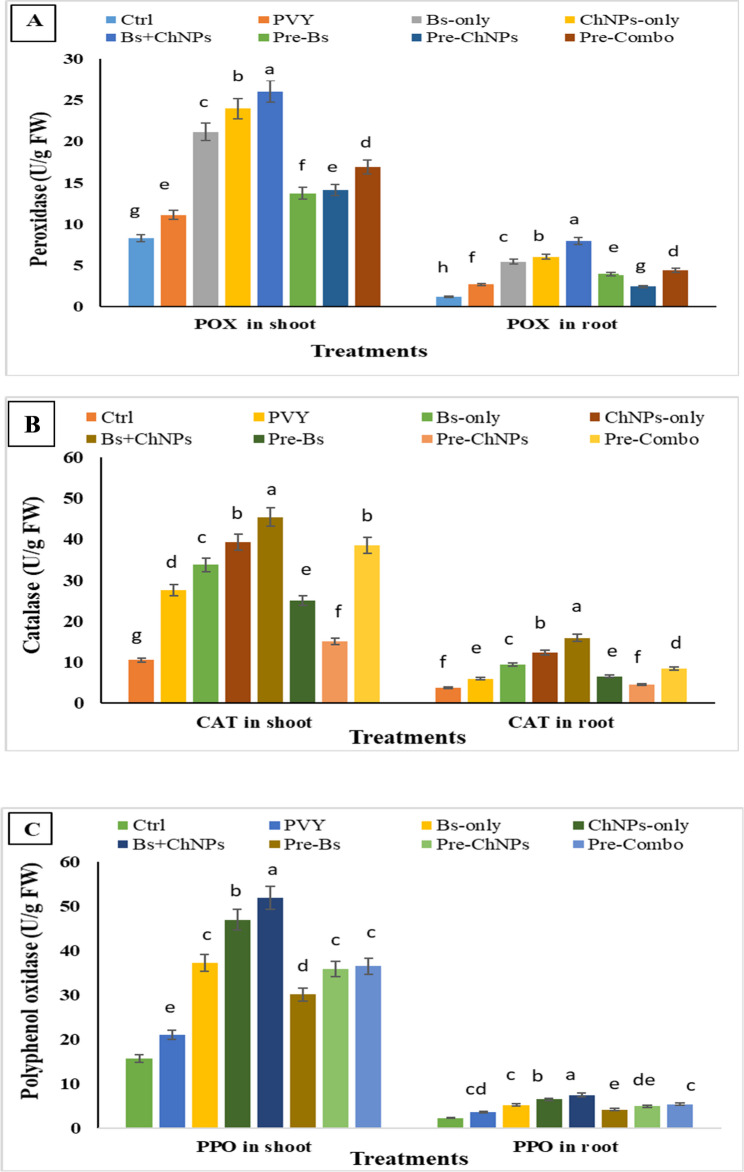
Effect of ChNPs, *B. subtilis* and combination on enzymatic antioxidants (**A**: peroxidase, **B**: catalase, and **C**: polyphenol oxidase) in shoots and roots of tomato plants under PVY infection. The average values (SD) for each treatment were determined from three replications. Means that are labeled with different letters in each bar indicate significant differences based on Duncan's Multiple Range Test at *p* < 0.05

Enzymatic antioxidant activity showed significant differences across treatments (*p* < 0.05), revealing the extent to which treatments modulated the oxidative defense system in tomato plants (Fig. 6A-C). In the control group, peroxidase (POX), catalase (CAT), and polyphenol oxidase (PPO) activities were low in both shoot and root tissues, with shoot POX at 8.31, CAT at 10.49, and PPO at 15.69 U/g FW. These values reflect baseline enzymatic status in unstressed plants. PVY infection triggered a moderate elevation in enzyme activities compared to control, likely as a stress-induced response. Shoot and root POX rose about 33.9% and 126.0%, CAT about 163.3%, 58.5%, and PPO about 34.2% and 61.5%, respectively, but these increases remained significantly below the levels achieved with biostimulant treatments.

Among healthy, unstressed plants treated with biostimulants, the combined application of *B. subtilis* and chitosan nanoparticles led to the strongest activation of all three enzymes. Shoot activities for POX, CAT, and PPO increased about 213.6%, 333.0%, and 230.4%, respectively, also root levels followed the same pattern. Chitosan alone also significantly elevated enzyme activities, followed by *Bacillus* alone, which was less effective than the combined treatment but still markedly higher than the control. Priming treatments applied before PVY exposure showed varying outcomes. The pre-combination group produced intermediate enzyme levels, increasing shoot and root POX by about 51.9%, 63.0%, CAT about 39.6%, 41.3%, and PPO by about 73.6%, 47.7% respectively, higher than PVY but lower than direct biostimulant application. The chitosan-primed plants showed modest improvement. These differences indicate that while priming offered partial restoration of enzymatic defense, it was less effective than full biostimulant application in healthy plants.

### Changes in osmolyte accumulation

Osmolyte accumulation parameters, namely free amino acids, proline, soluble sugars, and proteins, exhibited significant differences across treatments (*p* < 0.05), reflecting plant responses to viral stress and biostimulant interventions (Table 5). In the control group, levels of total free amino acids in shoots and roots were relatively low (3.45 and 0.18 mg/g FW), while proline was measured at 3.56 and 1.09 µmol/g FW, respectively. Soluble sugars and proteins in both tissues followed a similar trend, with shoot sugar content at 3.86 mg/g and soluble protein at 76.93 mg/g FW.

**Table 5:** Effect of ChNPs, *B. subtilis* and combination on osmolyte in shoots and roots of tomato plants under PVY infection

Treatments	Total free amino acids in shoot (mg glycine/g FW)	Total free amino acids in root (mg glycine/g FW)	Proline in shoot (µmol/g FW)	Proline in root (µmol/g FW)	Total soluble sugar in shoot (mg/g)	Total soluble sugar in root (mg/g)	Total soluble protein in shoot (mg/g)	Total soluble protein in root (mg/g)
Ctrl	3.447 ± 0.26^ g^	0.182 ± 0.06^d^	3.557 ± 0.30^f^	1.090 ± 0.17^ g^	3.857 ± 0.51^f^	1.083 ± 0.02^e^	76.933 ± 1.67^e^	0.962 ± 0.02^c^
PVY	3.907 ± 0.16^ g^	0.213 ± 0.04^d^	5.024 ± 0.12^e^	1.151 ± 0.06^ g^	4.873 ± 0.21^e^	2.052 ± 0.05^de^	30.533 ± 0.31^ g^	0.360 ± 0.02^e^
Bs-only	5.760 ± 0.16^f^	0.231 ± 0.03^d^	9.788 ± 0.44^b^	3.286 ± 0.04^e^	5.344 ± 0.24^e^	2.668 ± 0.23^d^	170.400 ± 3.86^c^	1.427 ± 0.02^b^
ChNPs-only	10.820 ± 0.64^b^	0.511 ± 0.02^ab^	5.188 ± 0.19^de^	4.310 ± 0.03^c^	9.220 ± 0.28^c^	5.243 ± 0.92^b^	188.000 ± 7.23^b^	1.482 ± 0.03^b^
Bs + ChNPs	14.733 ± 0.15^a^	0.548 ± 0.01^a^	15.197 ± 0.16^a^	5.049 ± 0.22^a^	13.228 ± 0.46^a^	6.932 ± 0.66^a^	218.000 ± 6.80^a^	2.247 ± 0.31^a^
Pre-Bs	6.617 ± 0.28^e^	0.363 ± 0.02^c^	5.706 ± 0.4^d^	2.612 ± 0.02^f^	8.542 ± 0.33^d^	3.811 ± 0.56^c^	42.933 ± 0.23^f^	0.636 ± 0.04^d^
Pre-ChNPs	7.217 ± 0.30^d^	0.470 ± 0.04^b^	7.166 ± 0.16^c^	3.829 ± 0.06^d^	10.686 ± 0.32^b^	4.786 ± 0.62^bc^	97.867 ± 4.60^d^	1.380 ± 0.01^b^
Pre-Combo	8.593 ± 0.28^c^	0.550 ± 0.09^a^	9.711 ± 0.42^b^	4.839 ± 0.09^b^	11.110 ± 0.12^b^	5.221 ± 0.71^b^	164.800 ± 2.88^c^	2.111 ± 0.02^a^

The average values (SD) for each treatment were determined from three replications. Means that are labeled with different letters in each bar indicate significant differences based on Duncan's Multiple Range Test at *p* < 0.05

PVY infection led to mild elevations in total amino acids and proline by about 13.3%, 41.2% in shoots and 17.0%, 5.6% in roots, respectively, total soluble sugars content was significantly increased in both shoot about 26.3% and root about 89.5%, while total soluble protein content was significantly decreased in both shoot about 60.3% and root about 62.5%, compared to the control, suggesting stress-induced osmotic adjustment. However, this response was not sufficient to maintain metabolic function, as reflected in the reduced protein contents across tissues.

Application of biostimulants to uninfected plants significantly enhanced osmolyte accumulation. The combined treatment of *B. subtilis* and chitosan nanoparticles led to significant increase (*p* < 0.05) of free amino acids in shoots and roots by about 327.4%, 201.1%, proline (327.2%, 363.2%), soluble sugars (243.0%, 540.1%), and proteins (183.4%, 134.1%), respectively over in control groups. Chitosan nanoparticles alone also induced notable increases in these compounds. In contrast, *Bacillus*-only treatments showed moderate elevation, with values often lower than chitosan-based applications.

Among primed plants, the combination of both agents before infection (pre-combination) demonstrated a strong recovery capacity. Amino acids and proline levels increased about 119.9%, 93.3% in shoots and 158.2%, 320.4% in roots, respectively, while sugars and proteins in shoots (128.0%, 614.0%) and roots (154.4%, 486.4%) were markedly higher than PVY-infected plants, respectively, indicating enhanced osmotic regulation. Pre-treatment with chitosan alone also improved osmolyte levels, though to a lesser extent. Pre-Bacillus treatment showed limited impact, with lower levels of all osmolytes, particularly in roots.

### Changes in yield and fruit quality

Yield traits varied significantly across treatments (*p* < 0.05), revealing the strong impact of PVY infection and the role of biostimulants in restoring productivity (Table 6). Control plants produced 2.05 kg of fruit with 25 fruits and a total size of 1272.20 cm^2^, establishing a baseline for comparison. PVY infection led to severe yield reductions: fruit mass dropped to 0.68 kg, with only 9 fruits and 186.60 cm^2^ in size. Despite the loss, the harvest index (0.128) was statistically similar to the control (0.120), suggesting proportional allocation to fruit biomass remained unchanged under stress.

**Table 6:** Effect of ChNPs, *B. subtilis* and combination on yield and fruits components of tomato plants under PVY infection

Treatments	No. fruits/plant	Yield (Kg)	Total fruits size Cm^2^/plant	Harvest Index	Carotenoid (mg/g FW)	Lycopene (mg/ml)	Total phenol (µg tannic acid/g)	Ascorbic acid (µg/g FW)	DPPH (mg/ml)	Anthocyanin (g/ml FW)
Ctrl	25.0 ± 0.71^e^	2.045 ± 0.21^d^	1272.2 ± 241.11^c^	0.120 ± 0.01^b^	5.51 ± 0.32^b^	14.371 ± 1.30^b^	1.44 ± 0.19^e^	0.61 ± 0.05^d^	0.29 ± 0.03^ h^	4.48 ± 0.32^ g^
PVY	9.0 ± 1.41^f^	0.68 ± 0.19^f^	186.6 ± 59.08^d^	0.128 ± 0.03^b^	1.80 ± 1.16^d^	3.114 ± 0.35^d^	2.04 ± 0.17^d^	0.92 ± 0.09^d^	1.52 ± 0.17^ g^	7.94 ± 0.23^f^
Bs-only	35.4 ± 0.55^c^	2.54 ± 0.23^c^	1713.0 ± 237.18^b^	0.125 ± 0.01^b^	3.43 ± 2.92^bcd^	8.586 ± 6.56^c^	2.28 ± 0.20^ cd^	1.21 ± 0.04^d^	2.73 ± 0.00^e^	18.48 ± 0.22^c^
ChNPs-only	39.4 ± 0.55^b^	3.05 ± 0.12^b^	2320.8 ± 300.74^a^	0.072 ± 0.00^d^	5.27 ± 0.11^bc^	20.626 ± 2.12^a^	2.48 ± 0.21^bc^	2.97 ± 0.82^b^	5.71 ± 0.11^b^	22.26 ± 0.73^a^
Bs + ChNPs	45.4 ± 3.05^a^	3.77 ± 0.53^a^	2440.8 ± 480.63^a^	0.052 ± 0.01^e^	8.64 ± 0.31^a^	22.091 ± 2.21^a^	3.66 ± 0.12^a^	4.08 ± 0.50^a^	6.74 ± 0.05^a^	20.37 ± 0.85^b^
Pre-Bs	24.2 ± 0.45^e^	1.65 ± 0.17^e^	1262.0 ± 110.08^c^	0.126 ± 0.01^b^	3.16 ± 0.13^ cd^	8.738 ± 0.15^c^	2.28 ± 0.3^ cd^	2.18 ± 0.52^c^	2.34 ± 0.03^f^	12.45 ± 0.28 e
Pre-ChNPs	29.2 ± 0.45^d^	2.51 ± 0.23^c^	1661.2 ± 117.45^b^	0.158 ± 0.02^a^	4.81 ± 0.10^bc^	13.443 ± 1.63^b^	2.55 ± 0.13^bc^	2.59 ± 0.30^bc^	3.35 ± 0.09^d^	12.49 ± 0.41^e^
Pre-Combo	34.8 ± 0.45^c^	3.10 ± 0.07^b^	1874.0 ± 103.89^b^	0.100 ± 0.00^c^	5.17 ± 0.02^bc^	15.294 ± 0.50^b^	2.70 ± 0.02^b^	3.72 ± 0.20^a^	4.60 ± 0.37^c^	13.61 ± 0.05^d^

The average values (SD) for each treatment were determined from three replications. Means that are labeled with different letters in each bar indicate significant differences based on Duncan's Multiple Range Test at *p* < 0.05

Among healthy, non-infected plants, combined treatment with *Bacillus subtilis* and chitosan nanoparticles resulted in the highest yield (3.77 kg), fruit number (45), and fruit size (2440.80 cm^2^). However, this increase came with a lower harvest index (0.052), indicating greater total biomass. Chitosan and *Bacillus* alone also improved yield (3.05 and 2.54 kg), with moderate fruit numbers (39 and 35) and harvest indices of 0.072 and 0.125, respectively. Priming before infection showed variable outcomes. The combined priming restored yield to 3.10 kg and fruit number to 35, closely matching biostimulant-only treatments. However, the harvest index remained moderate (0.100). Priming with chitosan alone provided partial recovery (2.51 kg), while *Bacillus* priming was less effective (1.65 kg), though still significantly better than PVY-infected plants (*p* < 0.05).

Fruit quality traits followed a similar trend. The control group showed moderate carotenoid (5.51 mg/g), lycopene (14.37 mg/ml), phenol (1.44 µg/g), and ascorbic acid (0.61 µg/g) levels, with low DPPH activity (0.29 mg/ml) and anthocyanins (4.48 g/ml). PVY infection reduced all compounds except DPPH, which increased to 1.52 mg/ml, likely as a stress response.

In healthy, treated plants, combined biostimulants led to the highest levels of carotenoids (8.64 mg/g), lycopene (22.09 mg/ml), phenols (3.66 µg/g), and ascorbic acid (4.08 µg/g), alongside high DPPH (6.74 mg/ml) and anthocyanins (20.37 g/ml). Chitosan alone yielded similarly strong results. *Bacillus* treatment enhanced anthocyanins (18.48 g/ml) but was less consistent for other markers.

Priming with both agents (Pre-Combo) partially restored fruit quality: lycopene (15.27 mg/ml) and carotenoids (5.16 mg/g) neared control levels, while phenols (2.70 µg/g) and ascorbic acid (3.60 µg/g) significantly improved over PVY. Pre chitosan nanoparticles and pre-Bacillus showed lower antioxidant recovery, especially in DPPH and anthocyanins. To evaluate the physiological and biochemical responses of tomato plants to PVY infection and the mitigating effects of different treatments, a comprehensive chemometric analysis was conducted. The dataset included 48 parameters encompassing growth traits, photosynthetic pigments, oxidative stress markers, antioxidant enzyme activities, non-enzymatic antioxidants, and fruit quality indicators. Eight treatment groups were compared: Ctrl (healthy control), PVY (infected untreated), Bs-only, ChNPs-only, Bs + ChNPs, and three pre-infection treatments—Pre-Bs, Pre-ChNPs, and Pre-Combo.

### Heatmap analysis

**Fig. 7:**
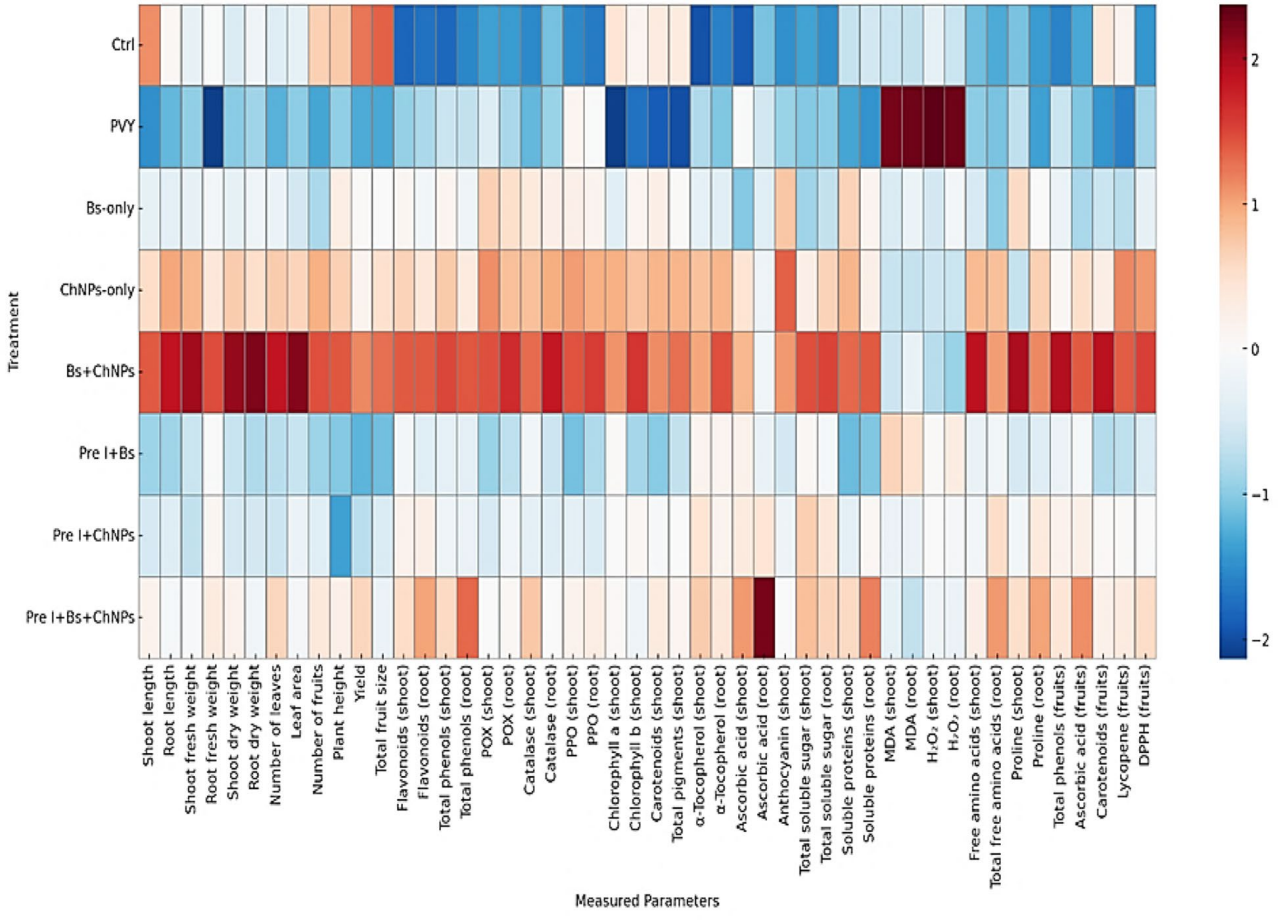
Hierarchical clustering of different treatments based on a heatmap generated using the z-score values

The heatmap provided an overview of how each treatment influenced the measured parameters (Fig. 7). The PVY group showed a widespread decline across most traits, particularly those related to growth (e.g., shoot length, root length, fresh and dry weights), photosynthetic pigments (chlorophylls a and b, carotenoids), and oxidative stress indicators (elevated MDA and H_2_O_2_).

In contrast, the Pre-Combo treatment (*B. subtilis* + chitosan nanoparticles applied after infection) demonstrated marked improvement across all functional categories. This group exhibited consistently higher values in antioxidant capacity (ascorbic acid, flavonoids, total phenols, DPPH), pigment content, growth parameters, and fruit quality traits (lycopene, fruit size, yield). The Pre-ChNPs and Pre-Bs treatments produced moderate improvements, suggesting partial mitigation of viral stress. Pre-infection treatments (Bs + ChNPs, Bs-only, ChNPs-only) showed inconsistent results, indicating that post-infection application was more effective in promoting recovery.

**Fig. 8:**
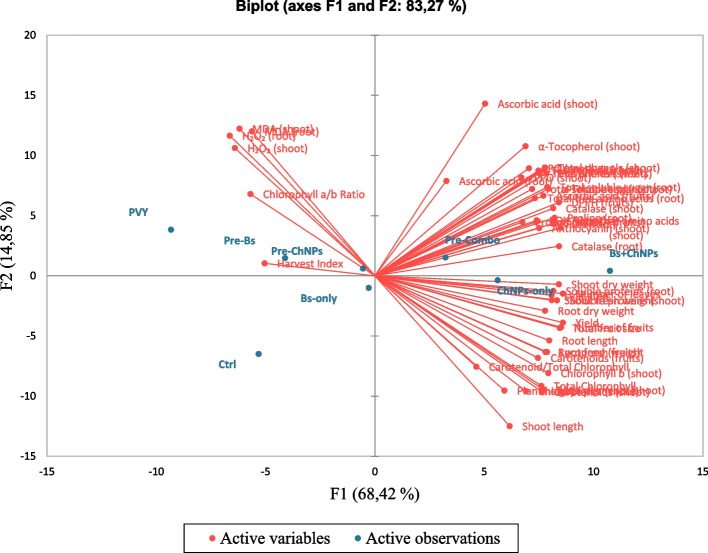
The chemometric evaluation using Principal Component Analysis (PCA) and hierarchical clustering provided further insights into the physiological and biochemical responses of tomato plants to different treatment regimens

The chemometric evaluation using Principal Component Analysis (PCA) and hierarchical clustering provided further insights into the physiological and biochemical responses of tomato plants to different treatment regimens (Fig. 8). These included control (Ctrl), PVY-infected plants (PVY), stimulant-treated plants (Bs-only, ChNPs-only, Bs + ChNPs), and pre-primed groups (Pre-Bs, Pre-ChNPs, Pre-Combo). The PCA biplot accounted for a total of 83.27% of the variance across the first two components (PC1 = 68.42%, PC2 = 14.85%), suggesting a strong discriminatory power among treatment profiles and their associated traits.

PVY-infected plants appeared isolated in the far negative region of PC1 and moderately positive along PC2. Their position was closely associated with stress indicators such as MDA (shoot/root) and H₂O₂ (shoot/root), supporting the interpretation that viral infection induced pronounced oxidative stress and damage. This placement is consistent with PVY-induced disruption of cellular homeostasis, suppression of pigment biosynthesis, and degradation of antioxidant defense systems, effects that have been well-documented in literature concerning systemic viral infections.

On the opposite end of PC1, Bs + ChNPs treatment emerged as the most distantly separated and positively aligned treatment, clustering tightly with beneficial variables including chlorophyll a and b, carotenoids, total pigments, ascorbic acid (shoot/root), α-tocopherol, flavonoids, proline, and total soluble sugars and proteins. The alignment of Bs + ChNPs with both photosynthetic and antioxidant traits suggest a coordinated activation of defense metabolism and physiological recovery mechanisms. Notably, this group showed improved association not only with primary metabolic markers but also with traits indicative of enhanced fruit quality and yield potential, such as lycopene, DPPH, and total phenols (fruit).

**Fig. 9:**
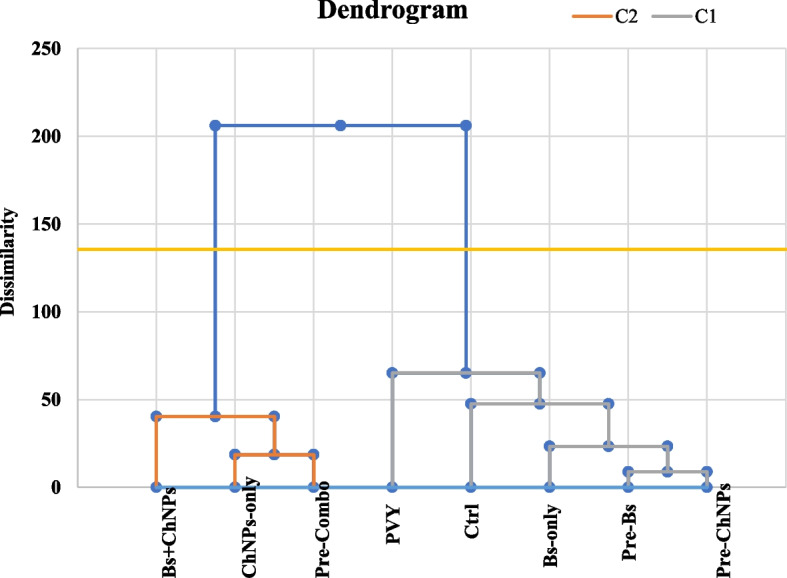
Cluster analysis of different treatments

ChNPs-only and Pre-Combo treatments also occupied positions on the positive side of PC1, clustering near Bs + ChNPs and exhibiting strong affinity with similar biochemical traits (Fig. 9). Although slightly less extreme in their enhancement profile, their alignment with key antioxidant and metabolite traits reinforces their functional impact on plant defense and recovery. Pre-Combo in particular demonstrated proximity to a broad spectrum of positive traits, suggesting that the pre-infection priming approach can effectively precondition plants to better resist or recover from viral stress.

In contrast, the control group (Ctrl) was located near the central axis of PC1 but appeared shifted downward along PC2. This reflects a moderate or baseline expression level across most traits without either strong activation or suppression. Interestingly, the Bs-only treatment—although applied in the absence of infection—remained close to the control, indicating limited physiological deviation. Its lack of alignment with antioxidant or growth-promoting traits suggests that *Bacillus subtilis* alone, under non-stressed conditions, may not sufficiently induce significant changes in plant performance.

Pre-Bs and Pre-ChNPs were positioned between PVY and the high-performing cluster, showing only partial recovery or defense activation. While these priming treatments offered some separation from the infected profile, they did not cluster with the most favorable trait groupings. This may imply that, in isolation, the priming effect of a single elicitor (either microbial or nano-based) is insufficient to induce the breadth of systemic protection needed to fully counter viral damage.

Hierarchical clustering supported these multivariate patterns by separating treatments into two distinct clusters. Cluster 1 included PVY, Ctrl, Bs-only, Pre-Bs, and Pre-ChNPs—treatments characterized by either minimal enhancement or profiles still impacted by stress. Within this cluster, the proximity of PVY and Pre-Bs highlights the limited protective effect achieved by *B. subtilis* priming alone. Similarly, Pre-ChNPs and Ctrl remained close, suggesting minimal physiological benefit in uninfected plants treated solely with chitosan nanoparticles.

In contrast, Cluster 2 included Bs + ChNPs, ChNPs-only, and Pre-Combo—treatments with the most enhanced physiological and biochemical signatures. These were closely associated with traits reflecting pigment stabilization, improved antioxidant content, and metabolic recovery. The tight grouping of Bs + ChNPs and ChNPs-only suggests that chitosan played a major role in mediating defense activation, while the combination with *B. subtilis* further boosted trait performance. The presence of Pre-Combo in this high-performing cluster supports the conclusion that pre-infection application of combined elicitors can produce results comparable to, or even approaching, those of post-stress combination treatments.

Collectively, these results highlight several important conclusions. First, viral infection induces a clear and measurable shift in plant physiology, particularly elevating stress biomarkers and suppressing pigments and metabolic products. Second, the application of biostimulants—especially in combination—offers a powerful strategy for either restoring physiological balance (in the case of Bs + ChNPs) or preparing plants in advance for viral stress (Pre-Combo). Third, treatments relying on a single agent (Bs-only, Pre-Bs, or Pre-ChNPs) provide limited protection and fail to redirect the plant’s profile toward optimal physiological performance.

The integration of PCA and clustering confirms that only treatments leveraging synergistic elicitor effects achieve widespread trait recovery. From a biological perspective, these combinations likely engage both the salicylic acid-dependent systemic acquired resistance (SAR) and the jasmonic acid/ethylene-dependent induced systemic resistance (ISR), resulting in broader and more effective defense activation. Such findings advocate for the strategic use of multi-agent priming as a preventive measure in crop management systems threatened by viral pathogens like PVY.

## Discussion

The production of ChNPs has been conducted using a variety of methods. When selecting an appropriate process for preparing ChNPs, it is essential to consider their stability, safety, and particle size. Biofabrication of ChNPs offers several advantages, including being a one-step process that is environmentally friendly, non-toxic, and energy-efficient. Additionally, biosynthesized ChNPs tend to be more stable [61]. ChNPs have been biosynthesized in a green manner using plant extracts, such as those from tomatoes [38]. The secondary metabolites found in the aqueous extracts of tomato fruits serve as reducing agents in the biofabrication of nanoparticles [38]. These biomolecules can function as both reducing and capping agents [38].

In this investigation, we employed a strategy for the cost-effective, eco-friendly, and biosafe synthesis of nanoparticles utilizing tomato fruit extract. Lycopene and other natural compounds found in tomatoes are used as capping and reducing agents in the green production of nanoparticles using tomato fruit extracts. Usually, this procedure combines a metal salt solution with tomato extract to create nanoparticles. According to Mansur and Yahya [62], tomato extract serves as a capping agent, stabilizing the nanoparticles and preventing their aggregation, as well as a reducing agent, turning metal ions into nanoparticles.

The size and shape of nanoparticles significantly influence their antimicrobial activity against microbial pathogens. Smaller particles can easily penetrate the cell walls of microorganisms, enhancing the uptake of the nanoparticles into microbial cells [38]. Scanning electron microscopy (SEM) analysis shows that the nanoparticles are spherical with smooth surfaces, although some are found in an agglomerated state. Transmission electron microscopy (TEM) analysis of the biosynthesized ChNPs reveals that their sizes range from 12.0 to 39.4 nm, confirming their spherical shape. The results indicate that ChNPs have a highly porous surface, attributed to their tendency to agglomerate. This porous nature and agglomeration capability make ChNPs valuable as a chitosan-based bio-nanopesticide [63]. Agglomeration is considered a primary phenomenon in the synthesis of novel ChNPs for agricultural applications [63]. Additionally, their porous structure can trap quenching molecules that effectively adsorb harmful chemicals in soil. In a study by Agarwal et al. [64], the sizes of ChNPs produced from chitosan and tripolyphosphate (TPP) were reported to range from 168 to 682 nm.

The elemental composition of ChNPs, as shown by the EDX spectra, is mostly constituted of carbon, oxygen, sodium, and phosphorus, with trace amounts of Mg, Al, Si, K, Ca, and Fe. Additionally, this study's elemental proportion of ChNPs is comparable to that of Abdallah et al. [38], who discovered that the elemental composition of ChNPs is made up of phosphorus (5.29%), oxygen (40.33%), and carbon (54.38%). Similar findings were also reported by Sotelo-Boyás et al. [65], who discovered that the EDX results verified the presence of carbon (72.63% atomic weight), oxygen (23.18%), nitrogen (3.20%), and additional elements like Na, Cl, K, and Ca in trace amounts.

Surface effects are strongly influenced by nanoparticle dispersion. Agglomeration occurs due to attractive interactions between particles, reducing their surface area and nanoscale characteristics. Electronic repulsion among particles affects their stability, with a higher zeta potential indicating greater stability [66]. ChNPs prepared by nanoprecipitation exhibited a zeta potential of + 36.5 mV, consistent with Elsahhar et al. [32], who reported 25.9 mV. DLS analysis showed that ChNPs had a size of 198.2 nm. Kheiri et al. [67] noted that positive zeta potentials result from residual protonated amine groups. To maintain stability through electrostatic repulsion, a zeta potential of at least ± 30 mV is necessary [68] ChNPs are less stable if their zeta potential is below + 30 mV [69].

XRD analysis is useful for determining a sample's crystalline structure based on its physical characteristics. Because of its amorphous form, chitosan exhibits a hump peak that appears at 2θ within the range of 20–30º [66] Three prominent peaks were found at 2θ = 20.4, 26.4, and 29.5° in the chitosan XRD patterns [70, 71] reported that the ChNPs showed diffraction peaks at 2θ = 10° and 20°. These peaks showed that there was a high degree of crystallinity in the chitosan. According to El-Naggar et al. [66], the displacement of crystalline peaks of chitosan may be the cause of the crystalline peaks at 19.67 and 20.84.

The stability of green nanoparticles may be partially attributed to the presence of capping proteins, which may also provide additional benefits as antimicrobial agents [38]. FTIR analysis of ChNPs reveals various functional groups, including hydroxyl (-OH), amine (-NH₂), and carbonyl (C = O) groups [72]. A characteristic peak at 1654.98 cm^−1^ in the FTIR spectrum of the chitosan standard indicates the vibrations associated with the carbonyl group, known as the amide band I [73]. Additionally, the FTIR spectrum of chitosan shows a peak at 895 cm^−1^, which corresponds to the stretching vibration of the saccharide moiety (C–O–C) [66]. The presence of these capping groups on the surface of ChNPs is confirmed by FTIR analysis. These groups help stabilize the ChNPs, preventing any agglomeration or aggregation that may occur in the colloidal phase [66].

Agarose gel electrophoresis analysis of PCR results showed that a particular band had been amplified by about 801 bp. According to Elsahhar et al. [32], electrophoresis of the RT-PCR product revealed an amplification of an 825 bp DNA fragment in plants infected with PVY, whereas no such amplification was identified in potato plantlets clear of PVY. These results are consistent with their findings.

Our results indicated that all treatments (ChNPs, *B. subtilis*, and their combination) significantly reduced virus accumulation, virus concentration, the percentage of infection, and disease severity (%DS) in treated tomato plants. Similar findings were reported for chitosan nanoparticles, which were shown to be an effective tool for preventing PVY disease in potato plants [32]. Additionally, ChNPs can bind to nucleic acids during virus penetration, causing damage to the virus. Chitosan also has the ability to inhibit the synthesis of mRNAs that encode for metabolic processes and infections caused by viruses or viroids [74]. ChNPs altered the integrity of the virus by producing incomplete and defective viral particles. A potential explanation for this is that the synthesized ChNPs possess a surface area with positive charges and positively charged amino groups on the chitosan glucosamine polymer chains. These properties suggest that ChNPs might exhibit high bio-reactivity, attracting viral RNA that contains negatively charged phosphate groups in its primary chain, which in turn suppresses virus replication and disease progression [29]. Moreover, since all viral proteins have negatively charged clusters of glycoproteins, it is reasonable to assume that the positively charged nanoparticles could also target the viral coat protein [29]. This supports the notion that the effectiveness of ChNPs in controlling plant viruses is strongly influenced by their nano-sized physicochemical properties, chemical nature, and bio-reactivity. The nano-sized form of chitosan appears to be crucial for its antiviral properties [29].

Several studies have highlighted the effectiveness of chitosan-based nanomaterials in controlling phytoviruses. When foliar-sprayed, these nanoparticles enter plant leaves through stomata or epidermal adsorption and are transported throughout the plant via the vascular system [31]. ChNPs inhibit the alfalfa mosaic virus by binding to its RNA, which disrupts replication [31]. Foliar application of ChNPs on *Nicotiana glutinosa* significantly decreased disease severity caused by the alpha mosaic virus (AMV), reduced virus accumulation, and increased the levels of antioxidant enzymes like POD, PR1, and PAL [28, 31]. Known for their non-toxic, biodegradable, and biocompatible properties, ChNPs act as effective biocontrol agents [32]. Chitosan may prevent microbial pathogen growth by disrupting cell membranes and interfering with biochemical processes, leading to plant defense responses [32]. Furthermore, ChNPs can enhance plant growth by improving nutrient uptake, photosynthesis, and cell division [32].

The use of PGPR to combat plant pathogens has gained recognition over the past two decades as a safe and effective alternative to traditional methods for managing plant viruses [75]. Previous studies have reported that the combination of chitosan and PGPR significantly reduces the severity of ToLCV infections [76, 77]. The effectiveness of ChNPs and *Bacillus* spp. may depend on the activation of specific genes in plants that enhance their resistance to viral infections. This mechanism involves modifying the plant's response to infection, which includes inhibiting the synthesis of virus-specific proteins, blocking the replication of viral RNA, and promoting the production of β-glucanase and chitinase. Additionally, it encourages processes such as lignification, callus formation, and increased activities of enzymes like polyphenol oxidase, peroxidase, and phenylalanine ammonia-lyase, along with the accumulation of phenolic compounds [75–77].

Our results indicated that all priming treatments significantly reduced the detrimental effects of PVY on vegetative biomass. This suggests that preemptively applying biostimulants, especially in combination, can help restore plant vigor under viral stress. PVY infection substantially decreased potato growth compared to healthy plants [6]. ChNPs mitigated stunted growth caused by the virus by lowering its infectivity, enhancing growth parameters beyond those of non-infected controls [29]. Chitosan is believed to boost plant growth by stimulating processes like nutrient uptake and photosynthesis [78]. Therefore, nano-based chitosan may effectively alleviate viral infections [29]. Additionally, foliar application of *B. subtilis* reduced disease severity after TMV infection, promoting tomato growth and decreasing viral accumulation [1]. The slower increase in symptoms in treated plants may be linked to inhibited viral movement [1]. *B. subtilis* enhances growth through various beneficial metabolites, while chitosan nanoparticles positively influence vegetative growth, yield, and mineral content [27].

Photosynthesis is vital in plants, and virus-plant interactions can disrupt pigment production in chloroplasts and inhibit photosystem II activity [6]. Our findings indicate that PVY drastically reduces pigment biosynthesis, but priming with biostimulants helps protect pigment integrity. This combined treatment effectively enhances pigment levels in both healthy and stressed plants. The decreased chlorophyll content and photosynthetic characteristics in PVY-infected leaves. Carotenoids, serving as accessory pigments, protect chlorophyll by preventing harmful oxygen species [6]. Treated plants exhibited higher chlorophyll and carotenoid levels than the control group, likely due to increased photosynthate levels [79].

Oxidative damage to cell membranes is a major harmful effect of viral infections [6]. Our findings confirm that PVY induces oxidative stress, but biostimulants, particularly in combination, effectively reduce ROS accumulation. Priming with these agents offers measurable protection, while direct application to uninfected plants yields the lowest stress levels. The increased levels of hydrogen peroxide, superoxide, hydroxyl radicals, and malondialdehyde in PVY-infected plants. In severely affected plants displaying symptoms like mosaic patterns and yellowing, these oxidative markers were notably elevated, suggesting oxidative damage to pigments [6]. Furthermore, the application of *B. subtilis* significantly lowered MDA and H_2_O_2_ levels compared to non-infected plants [1]. This reduction in oxidative stress enzyme activity helps maintain cell membrane integrity [80].

Our findings show that PVY significantly boosts the antioxidant response in tomato plants. The use of biostimulants, especially in combination, enhances non-enzymatic antioxidant reserves in both tissues, providing better protection and recovery while maintaining cellular redox balance under biotic stress. This aligns with Al-Mokadem et al. [6], who found that healthy control plants had higher levels of glutathione, ascorbic acid, and phenolics than inoculated plants. ChNPs enhance plant defenses against viruses like Bean Yellow Mosaic Virus (BYMV) by increasing PR-1 gene expression and the activity of defense-related enzymes [29]. The increase in total phenolic content is linked to the activity of phenylalanine ammonia lyase, crucial for producing phenolics that protect against pathogens [29]. Additionally, polyphenolic compounds serve as a defense mechanism in tomato plants treated with *Bacillus* sp. during TMV infection [1]. In both virus-infected plants and those treated with nanoparticles, the production of phenolic compounds rises, helping to scavenge ROS and increasing antioxidant levels [27]. ChNPs improve resistance against the Alfalfa Mosaic Virus in pepper plants by triggering cell signaling and enhancing protective mechanisms [27]. In addition, compared to TMV infection alone, *B. amyloliquefaciens* treatments significantly boosted ascorbic acid and non-enzymatic antioxidants in tomato tissues, which helps alleviate viral infection symptoms and inhibit RNA virus replication [80].

Viral infections produce ROS that can damage proteins, lipids, and DNA in plant cells [6]. In response, plants generate both non-enzymatic and enzymatic compounds to mitigate ROS and oxidative damage [81]. Our findings show that biostimulants, particularly in combination, significantly enhance antioxidant enzyme activities, surpassing the effects of priming or PVY-induced stress. In tomato plants treated with *B. subtilis* and infected by TMV, there was a notable increase in the activities of antioxidant enzymes, such as PPO, SOD, and POX, compared to infected plants [1]. POX aids in plant defense through lignin synthesis, creating a physical barrier against viral infection. These results support previous studies demonstrating that Bacillus species can enhance the activity of plant enzymes against ROS [1]. Moreover, the significant reduction in malondialdehyde and hydrogen peroxide levels underscores the effectiveness of Bacillus amyloliquefaciens in reducing oxidative stress in virus-infected plants. Increased antioxidative enzymes like POX and PPO also help limit tissue oxidation and pathogen penetration by strengthening cell walls [80].

The current results demonstrate the ability of combined biostimulant treatments to promote osmoprotectant accumulation, improving metabolic activity and stress tolerance. While PVY infection alone upset the osmolyte balance and hampered physiological performance, priming, especially when paired with other medicines, provided intermediate protection. Because ChNPs increase plant resistance against Alfalfa Mosaic Virus in pepper plants, treatments with these nanoparticles result in greater proline levels [27]. A decrease in proline oxidation to glutamate, an increase in protein turnover, or a decrease in protein consumption could all be responsible for the greater proline content. Additionally, it might trigger the genes involved in proline biosynthesis, which convert glutamate into proline.

The results indicate that combined biostimulants enhance yield and fruit quality while offering partial protection against viral stress. This dual benefit supports their use in improving reproductive performance and fruit nutritional quality. For instance, the foliar application of chitosan nanoparticles (0.1%, w/v) significantly boosts finger millet growth, yield, and mineral content [71]. Additionally, combining *Pseudomonas libanesis* with selenium nanoparticles (75 ppm) and *Bacillus thuringiensis* AZP2 with titanium dioxide nanoparticles has proven more effective for plant production than using PGPB or nanoparticles alone [82, 83]. PGPB benefits plants by fixing nitrogen, producing plant hormones and siderophores, and enhancing nutrient availability, particularly for phosphorus, potassium, and iron [84]. The production of indole-3-acetic acid (IAA) by PGPB increases root length and weight, improving nutrient uptake.

## Conclusion

The use of biosynthesized ChNPs and Bacillus significantly suppressed the PVY in tomato cultivation while also enhancing yield and its components. The combination of ChNPs and Bacillus proved to be a highly effective strategy, further improving plant health and the quality of the fruits. Both biosynthesized ChNPs and Bacillus can be considered environmentally friendly agents for controlling PVY in tomato crops. Additionally, these compounds can serve as valuable fertilizers that promote plant growth and increase antioxidant compounds in tomato fruits, thereby contributing to improved crop quality and human nutrition. ChNPs may function as elicitor molecules, activating plant immunity and triggering a hypersensitivity response through elevated antioxidant levels and defensive enzyme activity.

## Data Availability

All data generated or analyzed during this study are included in this published article.
